# Preliminary clinical analysis and pathway study of S100A8 as a biomarker for the diagnosis of acute deep vein thrombosis

**DOI:** 10.1038/s41598-024-61728-6

**Published:** 2024-06-10

**Authors:** Wenjie Zeng, Yangyang Gao, Qitao Wang, Junyu Chi, Ziyan Zhu, Qingfei Diao, Xin Li, Zhen Wang, Ming Qu, Yongquan Shi

**Affiliations:** 1 Graduate School, Hebei North University, Zhangjiakou, 075000 Hebei China; 2 Vascular Gland Surgery, The First Affiliated Hospital of Hebei North University, Zhangjiakou, Hebei China; 3 Department of Clinical Laboratory Center, Shandong Second Provincial General Hospital, Jinan, Shandong China

**Keywords:** Deep venous thrombosis, Diagnostic biomarkers, S100A8, Protein microarray chip, Embolism, Peripheral vascular disease, Thromboembolism, Thrombosis, Diagnostic markers

## Abstract

Herein, we aimed to identify blood biomarkers that compensate for the poor specificity of D-dimer in the diagnosis of deep vein thrombosis (DVT). S100A8 was identified by conducting protein microarray analysis of blood samples from patients with and without DVT. We used ELISA to detect S100A8, VCAM-1, and ICAM-1 expression levels in human blood and evaluated their correlations. Additionally, we employed human recombinant protein S100A8 to induce human umbilical vein endothelial cells and examined the role of the TLR4/MAPK/VCAM-1 and ICAM-1 signaling axes in the pathogenic mechanism of S100A8. Simultaneously, we constructed a rat model of thrombosis induced by inferior vena cava stenosis and detected levels of S100A8, VCAM-1, and ICAM-1 in the blood of DVT rats using ELISA. The associations of thrombus tissue, neutrophils, and CD68-positive cells with S100A8 and p38MAPK, TLR4, and VCAM-1 expression levels in vein walls were explored. The results revealed that blood S100A8 was significantly upregulated during the acute phase of DVT and activated p38MAPK expression by combining with TLR4 to enhance the expression and secretion of VCAM-1 and ICAM-1, thereby affecting the occurrence and development of DVT. Therefore, S100A8 could be a potential biomarker for early diagnosis and screening of DVT.

## Introduction

Venous thromboembolism (VTE) is the third most common cardiovascular and cerebrovascular disease after acute myocardial infarction and stroke^1^. The prevalence of VTE is increasing owing to factors such as the aging population^2^. VTE includes deep venous thrombosis (DVT) and pulmonary embolism (PE), which are manifestations of the same disease at different stages^3^. The main adverse events of DVT include limb swelling and pain, necrosis, PE, and post-thrombotic syndrome (PTS), which seriously affect the survival rate and quality of life of patients^1^. In recent years, blood biomarkers, such as P-selectin, E-selectin, particles, thrombin, factor VIII, fibrin monomers, and inflammatory cytokines, have been extensively explored for the diagnosis of DVT^4–9^. The diagnosis of DVT primarily relies on the single inspection of D-dimer, combined with imaging examinations^10,11^. Therefore, there is a lack of efficient and simple diagnostic methods.

S100A8, also known as MRP8, is a calcium-binding protein secreted as a heterodimer of S100A9 by immune cells and activated by necrotic cells and pathogen-related molecular patterns^12^. The secretion and expression of S100A8/S100A9 complex proteins have been associated with the activity of multiple inflammatory diseases^13,14^. During the inflammatory response, immune cells are activated and release inflammatory mediators to form a large regulatory network that mediates leukocyte migration to endothelial cells^15^. Viemann et al. demonstrated that S100A8/S100A9 secreted by phagocytes can specifically bind to endothelial cells in vitro, promoting the expression of most inflammatory factors and prothrombotic reactions^16^. Platelet-derived S100A9 directly regulates platelet function and arterial thrombosis by binding to CD36 receptors^17^. Wang et al. conducted extensive biochemical characterization and confirmed that mouse MRP-14 is functionally equivalent to its human counterpart and that S100A9 can directly induce DVT by inducing neutrophil extracellular traps^18^. However, the molecular mechanism of action of S100A8 in DVT has not been fully elucidated.

Toll-like protein 4 (TLR4) is a transmembrane signaling receptor expressed on the cell surface and is a specific endogenous ligand of S100A8, inducing various intracellular signal transduction pathways upon activation^19–21^. TLR4 activates the p38 mitogen-activated protein to produce a variety of pro-inflammatory factors that accelerate the inflammatory response. Intercellular adhesion molecule 1 (ICAM-1) and vascular cell adhesion molecule 1 (VCAM-1) are members of the immunoglobulin family of cell adhesion molecules (CAM) and constitute well-known markers of vascular function^22^. These molecules are expressed on induced endothelial cells and increase in the blood during an inflammatory process, participating in the inflammatory process and synergistically promoting the strong adhesion of leukocytes to endothelial cells^23,24^.

Therefore, in the current study, we screened the S100A8 protein using protein microarrays and explored the mechanism through which S100A8 induces the expression of VCAM-1 and ICAM-1 to promote DVT via the TLR4/p38MAPK signaling pathway. Our findings could provide novel insights into the pathogenesis and treatment of DVT.

## Results

### Basic information for patients with DVT

We collected blood from 40 patients with DVT (average age 60.73 ± 1.695 years; 19 men and 21 women) and 40 controls (average age 60.5 ± 1.737 years; 15 men and 33 women). Table 1 summarizes the characteristics of each group. No significant differences in age, sex, or body mass index (BMI) were observed between patients with DVT and the control group (*P* > 0.05). Furthermore, information regarding patient comorbidities and disease-related predisposing factors was collected(Supplemental Table 1).

**Table 1:** Basic information characteristics of patients.

	DVT Cases (n = 40)	Control (n = 40)	P value
Age, mean ± SD, years	60.73 ± 1.695	60.5 ± 1.737	0.926
Sex			0.119
Men, n (%)	19 (47.5)	15 (37.5)	
Women, n (%)	21 (52.5)	33 (82.5)	
Body mass index, mean ± SD, kg/m2	25.17 ± 0.59	24.65 ± 0.45	0.482
Smoking, n (%)	5 (12.5)	5 (12.5)	1.000
Alcohol consumption, g/day; n (%)	3 (7.5)	3 (7.5)	1.000
Hypertension, n (%)	14 (35)	6 (15)	0.039
Diabetes mellitus, n (%)	4 (10)	3 (7.5)	1.000
Risk factors Provoked			
Fracture (< 3 months), n (%)	10 (25)	11 (27.5)	0.799
Inflammation (< 3 months), n (%)	2 (5)	1 (2.5)	1.000
Operative injury (< 3 months), n (%)	1 (2.5)	0	1.000
Stroke (> 1 year), n (%)	4 (10)	4 (10)	1.000
Hypothyroidism, n (%)	1 (2.5)	0	1.000
Coronary heart disease, n (%)	2 (5)	1 (2.5)	1.000
Hyperlipidemia, n (%)	1 (2.5)	1 (2.5)	1.000

### Differential proteins between the DVT and control groups

**Figure 1:**
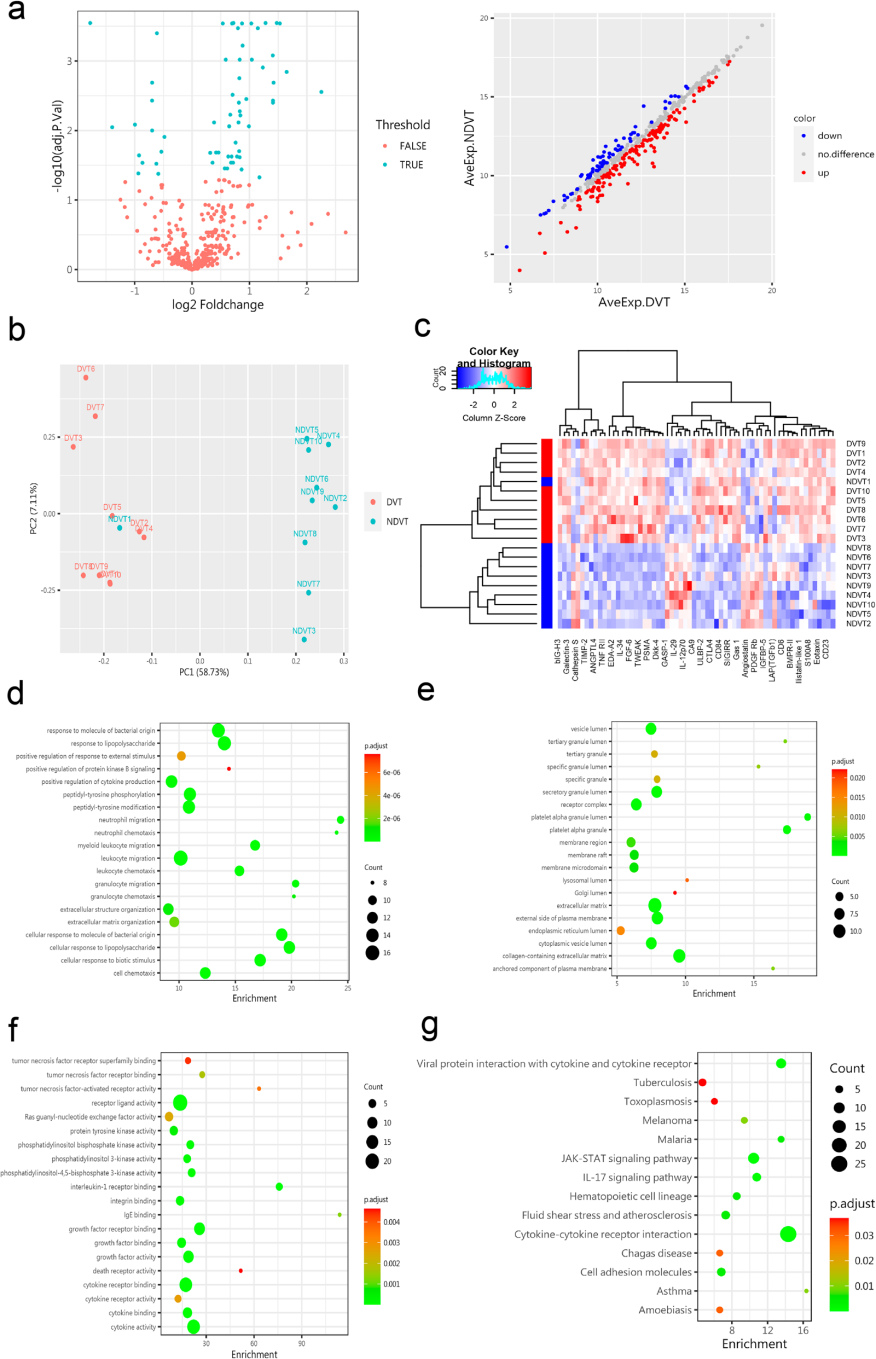
Screening for differential proteins through proteomics methods. (**a**) The green dots in the left plot represent 62 differential proteins (*P* < 0.05), the red dots represent undifferentiated proteins, and the blue dots in the right scatter plot represent directional proteins. Down indicates adjustment, red indicates upward adjustment, and gray indicates no difference. (**b**) The red dots in the PCA plot represent the deep venous thrombosis (DVT) group (n = 10), and the green dots represent the non-DVT (NDVT) group (n = 10). Inter-group analysis revealed significant differences (*P* < 0.05). (**c**) In the clustering heat map, the color depth of the red part represents the expression level of each upregulated differential protein in the DVT group, and the blue color represents the NDVT group. (**d**–**f**) The highest enrichment items of BP (biological process), CC (cell component), MF (molecular function), and Kyoto Encyclopedia of Genes and Genomes (**g**) for differential proteins.

Sixty-two significantly different proteins were screened between both groups using protein chip detection combined with GSH-CAA-440 data analysis software after adjusting the P value (adj.P.Val) to < 0.05 and the fold change to > 1.2 or < 0.83 (absolute logFC > 0.263) (Fig. 1a). Specifically, the first six significantly different proteins included LAP (TGFb1) (*P* < 0.001), Galectin-3 (*P* < 0.001), Gas 1 (*P* < 0.001), S100A8 (*P* < 0.001), CHI3L1 (*P* < 0.001), and FGF-6 (*P* < 0.001); additional detailed data differences are shown in Supplemental table 2. Significant differences were observed between the two groups in the principal component analysis (Fig. 1b), and differential proteins were visualized using clustering heat maps (Fig. 1c). Subsequently, we performed gene ontology (GO) and Kyoto Encyclopedia of Genes and Genomes (KEGG) pathway enrichment analyses using the DAVID online tool. GO analysis showed the highest enrichment in the "leukocyte migration" of BP (Biological Process) (Fig. 1d), the "extracellular matrix" of CC (Cell Component) (Fig. 1E), and the "receptor ligand activity" of MF (Molecular Function) (Fig. 1f). Based on the differential proteins in the KEGG 6.0 (http://www.kegg.jp) pathway analysis, "Cytokine-cytokine receptor interaction,” "JAK-STAT signaling pathway," and "IL-17 signaling pathway" may be involved in the process of DVT, as shown in Fig. 1g.

### S100A8, VCAM-1, and ICAM-1 levels in human plasma

**Figure 2:**
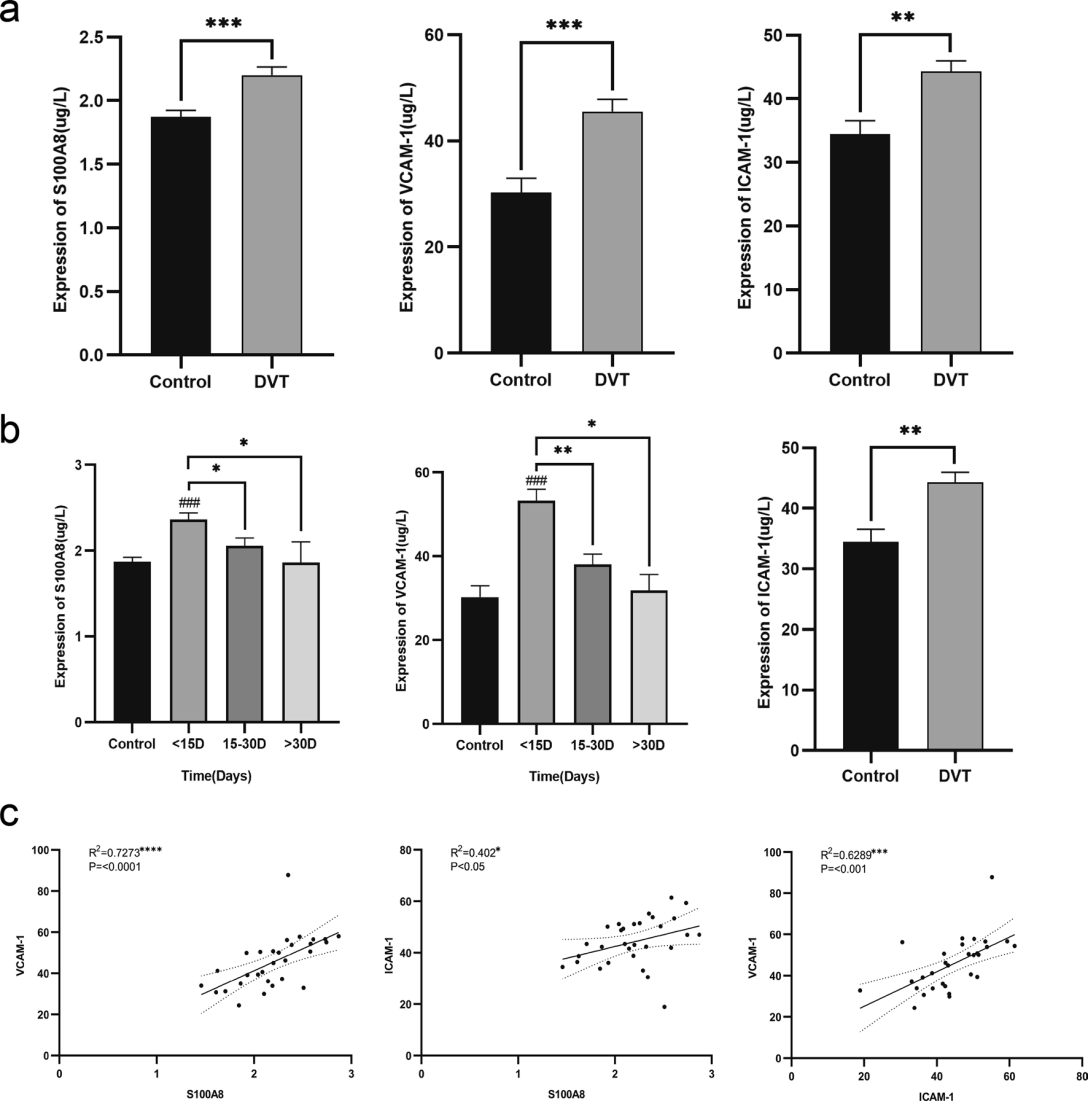
Patients with DVT have significantly higher plasma levels of S100A8, VCAM-1, and ICAM-1 than patients in the control group, and the levels positively correlate with each other (**a**) S100A8 (*P* < 0.001), VCAM-1 (*P* < 0.01), and ICAM-1 (*P* < 0.001) protein concentrations in plasma of the deep venous thrombosis (DVT) group (n = 30) and control group (n = 38) were detected using ELISA. Data are presented as mean ± standard deviation (SD). (**b**) Based on the data in A, DVT was divided into three groups according to the time of disease discovery: < 15 days (n = 16), 15–30 days (n = 11), and > 30 days (n = 13). Alterations in the levels of S100A8, VCAM-1, and ICAM-1 level can be observed. ^###^*P* < 0.001, ^##^*P* < 0.01, and ^#^*P* < 0.05 are compared with the control group, and ***P < 0.001, **P < 0.01, and *P < 0.05 are compared with data within 15 days, respectively. Data are mean ± SD. (**c**) According to the data in A, the correlation between S100A8, VCAM-1, and ICAM-1 in the plasma of patients with DVT. Data were analyzed using Spearman correlation analysis. ELISA, enzyme-linked immunosorbent assay; ICAM-1, intercellular adhesion molecule 1; VCAM-1, vascular cell adhesion molecule 1.

Plasma levels of S100A8 (*P* < 0.0001), VCAM-1 (*P* < 0.0001), and ICAM-1 (*P* < 0.0001) were significantly increased in the DVT group when compared with those in the control group (Fig. 2a, Supplemental table 3). DVT was classified based on the time of disease discovery. Comparisons were performed within 15 days (n = 16), between 15 and 30 days (n = 11), and after 30 days (n = 3). Plasma levels of S100A8 (*P* < 0.001) and VCAM-1 in patients with DVT onset within 15 days were compared. The levels of VCAM-1 (*P* < 0.001) and ICAM-1 (*P* < 0.001) were more notably elevated in patients with DVT onset within 15 days than in those who developed symptoms after 15 days (Fig. 2b), and the levels of these three proteins gradually decreased with time and returned to normal. Plasma S100A8 levels in the DVT group were positively correlated with VCAM-1 (R2 = 0.727, *P* < 0.0001) and ICAM-1 (R2 = 0.403, P = 0.027) levels. VCAM-1 levels were positively correlated with ICAM-1levels (R2 = 0.629, P = 0.0002) (Fig. 2c).

### Levels of TLR4, p38MAPK, ICAM-1, and VCAM-1 in human umbilical vein endothelial cells (HUVECs) after intervention

**Figure 3:**
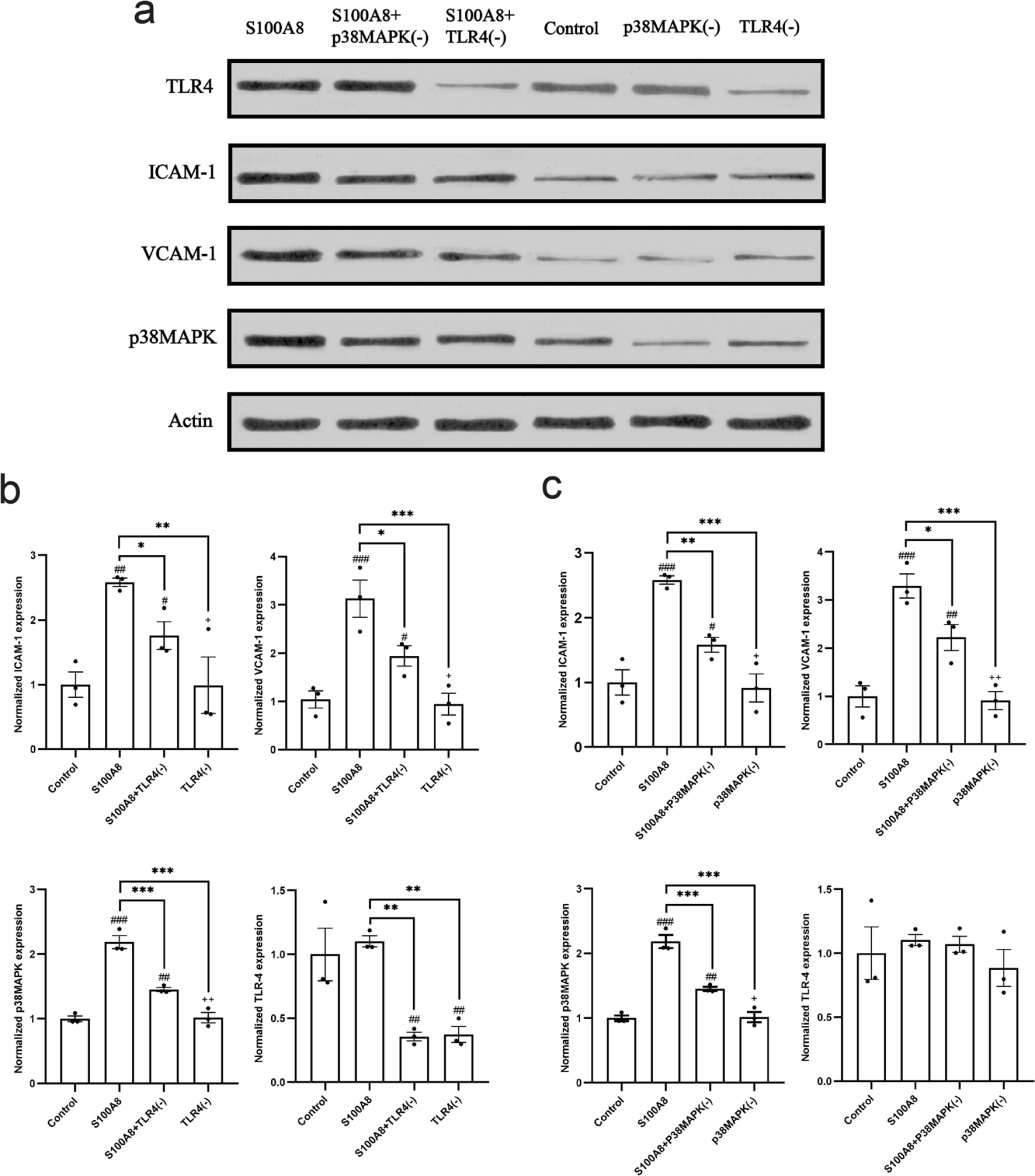
S100A8 can alter the expression of VCAM-1 and ICAM-1 in HUVECs via the TLR4/p38MAPK axis. (**a**) Human umbilical vein endothelial cells (HUVECs) were initially incubated with inhibitors for 30 min and then treated with exogenous S100A8 for 2 h. The levels of TLR4, p38MAPK, VCAM-1, and ICAM-1 in different environments were detected by western blotting. (**b**) Expression levels of protein TLR4, p38MAPK, VCAM-1, and ICAM-1 after blocking TLR4 protein. ^###^*P* < 0.001, ^##^*P* < 0.01, and ^#^*P* < 0.05 are compared with the control group, ***P < 0.001, **P < 0.01, and *P < 0.05 are compared with the simple S100A8 group, and ^+++^*P* < 0.001, ^++^*P* < 0.01, and ^+^*P* < 0.05 are compared with the TLR4 blocking group. (**c**) Expression levels of protein TLR4, p38MAPK, VCAM-1, and ICAM-1 after blocking the p38MAPK signaling pathway. ^###^*P* < 0.001, ^##^*P* < 0.01, and ^#^*P* < 0.05 are compared with the control group, ***P < 0.001, **P < 0.01, and *P < 0.05 are compared with the pure S100A8 group, ^+++^*P* < 0.001, ^++^*P* < 0.01, and ^+^*P* < 0.05 are compared with the p38MAPK blocking group. ICAM-1, intercellular adhesion molecule 1; VCAM-1, vascular cell adhesion molecule 1; TLR4, Toll-like protein 4.

Following treatment with the TLR4 inhibitor, TAK-242 ( +), the addition of exogenous S100A8 did not promote the expression of p38MAPK, VCAM-1, or ICAM-1 protein (Supplemental file 1). In HUVECSs, p38MAPK expression was increased in the group not pretreated with TAK-242 (-) (*P* < 0.001), VCAM-1 (*P* < 0.001), and ICAM-1 (*P* < 0.001) (Fig. 3a and b). Furthermore, the pretreatment of cells with p38MAPK inhibitor, SB203580 ( +), prior to exogenous S100A8 stimulation significantly reduced ICAM-1 (*P* < 0.001) and VCAM-1 (*P* < 0.001) levels when compared with cells untreated with SB203580 (-) (Fig. 3a and c).

### Changes in inflammatory cells and S100A8 levels in rat DVT tissues

**Figure 4:**
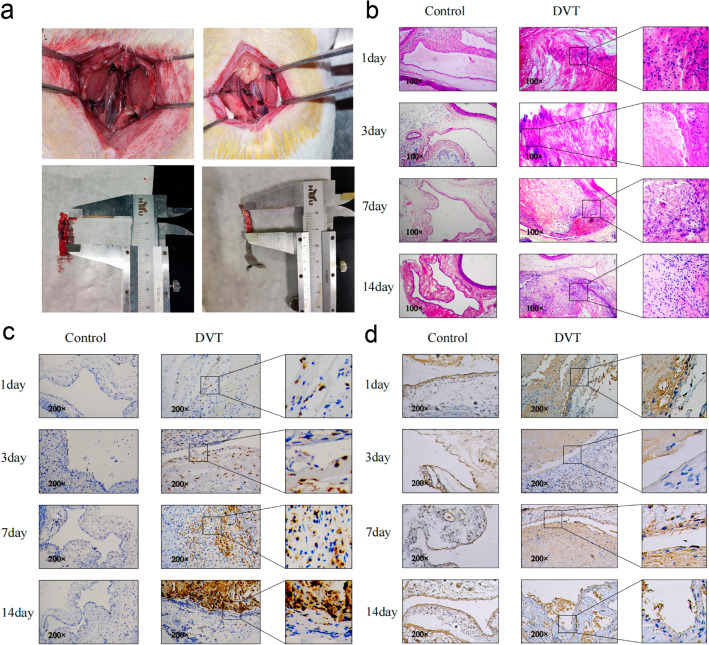
Changes in local inflammatory cell and S100A8 levels at four time points (1D, 3D, 7D, and 14D, respectively, after venous thrombosis). (**a**) Rat model of DVT induced by stenosis of the inferior vena cava and representative views of thrombosed veins and control veins. (**b**) Cross-section of hematoxylin and eosin stained blood vessel at 100 × magnification showing neutrophil accumulation in the thrombus. (**c**) Gradual changes in numbers of CD68-labeled monocyte macrophages shown with brown dye. (**d**) The vein wall is damaged, and some intact endothelial cells are stained brown by S100A8. 1D, day 1; 3D, day 3; 7D, day 7; 14D, day 14.

The DVT model was established, and the blocked lumen tissue appeared dark red (Fig. 4a and b). The lumen and vessel wall of the DVT group contained a large number of infiltrating neutrophils and a small number of lymphocytes on day 1 after thrombosis when compared with the control group, reaching a peak on day 3, and the thrombus began to organize simultaneously (Fig. 4b, Supplemental file 2). On day 7, CD68-positive mononuclear macrophages were observed in the vein wall and thrombus, indicating that inflammatory cells were gradually recruited as the formed thrombus over time (Fig. 4b and c, Supplemental file 3). However, CD68 expression in the DVT group was downregulated on days 1 and 3 when compared with the control group, and the levels of S100A8 in the vein wall gradually increased from day 1 to day 3, remaining the same as those in the control group thereafter (Fig. 4d, Supplemental file 4). The veins in the sham-operated group remained completely unobstructed, exhibiting limited inflammation. Levels of S100A8 in the vein wall increased with the decrease of inflammatory cells in DVT.

### Changes in S100A8, TLR4, p38MAPK, and VCAM-1 levels in the vein walls of DVT rats

**Figure 5:**
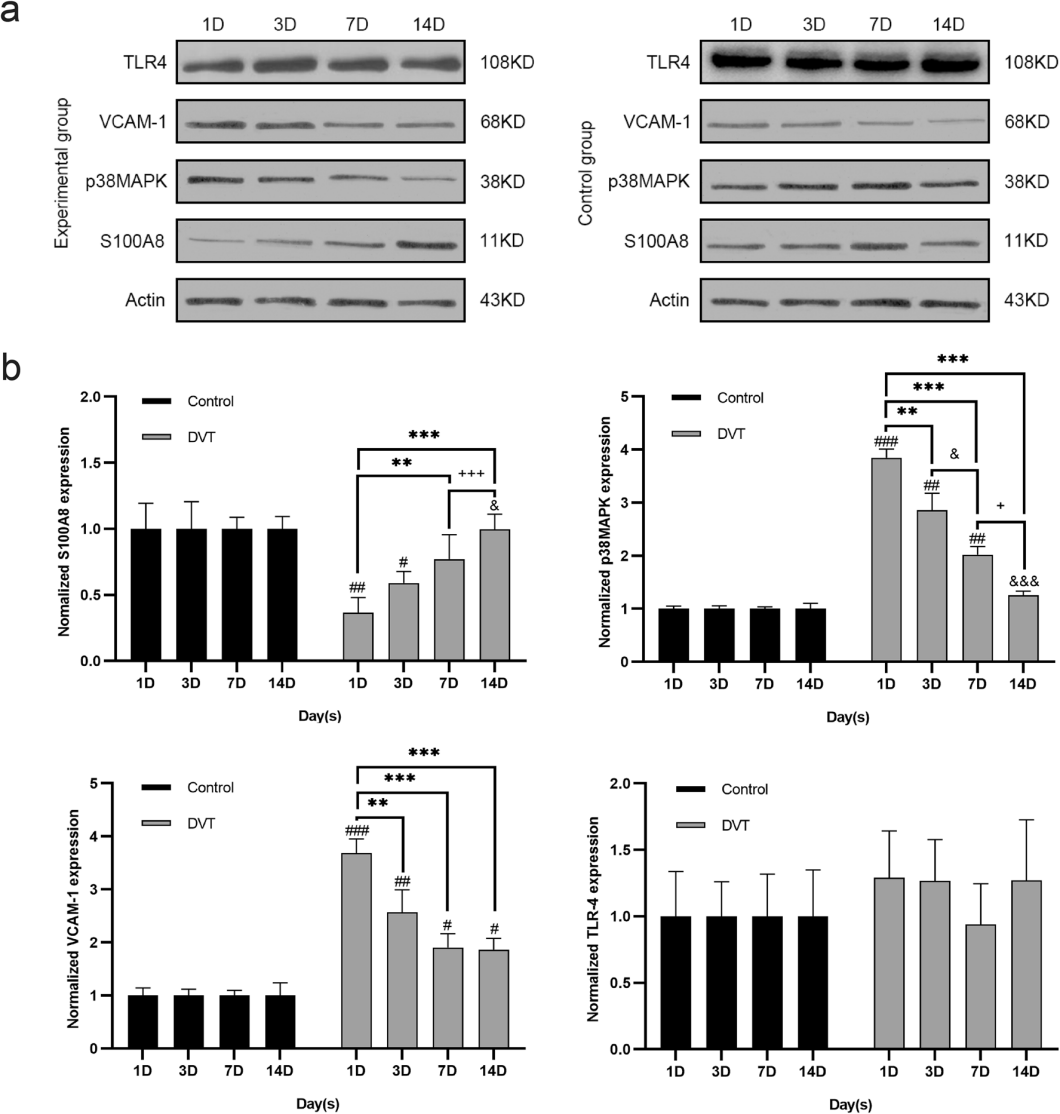
Expression levels of S100A8, TLR4, p38MAPK, and VCAM-1 protein in the vein walls at 1D, 3D, 7D, and 14D. (**a**) Western blotting results of S100A8, TLR4, p38MAPK, and VCAM-1 protein levels in the vein walls of rats in the experimental and control groups at four time points: day 1 (1D), day 3 (3D), day 7 (7D), and day 14 (14D). (**b**) Normalized expression of TLR4, VCAM-1, p38MAPK, and S100A8 in the vein wall of rats in the experimental and control groups. ^###^*P* < 0.001, ^##^*P* < 0.01, and ^#^*P* < 0.05 are compared with the control group. ***P < 0.001, **P < 0.01, and *P < 0.05 are compared with the 1D group. ^&&&^*P* < 0.001, ^&&^*P* < 0.01, and ^&^*P* < 0.05 are compared with the 3D group. ^+++^*P* < 0.001, ^++^*P* < 0.01, and ^+^*P* < 0.05 are compared with the 7D group. VCAM-1, vascular cell adhesion molecule 1; TLR4, Toll-like protein 4.

The vein walls of DVT rats were subjected to western blotting to further elucidate the relationship between S100A8 levels through the MAPK inflammatory pathway and ICAM-1 and VCAM-1 levels in the occurrence and development of DVT (Fig. 5a, Supplemental file 1). S100A8 significantly decreased from day 1 (*P* < 0.01), gradually increased from day 3, and returned to control levels on day 7 (Fig. 5b). Simultaneously, p38MAPK levels in DVT rats were significantly increased when compared with those in the control group from day 1 (*P* < 0.001) and gradually decreased thereafter, with a gradual increase in S100A8 in the vein wall; however, TLR4 levels did not differ significantly between the groups (Fig. 5b). VCAM-1 levels increased significantly on day 1 (*P* < 0.001) and decreased thereafter, with increasing S100A8 concentration (Fig. 5b), indicating that S100A8 activated p38MAPK expression, which, in turn, upregulated VCAM-1 expression after DVT.

### Expression levels of S100A8, ICAM-1, and VCAM-1 in rat plasma

**Figure 6:**
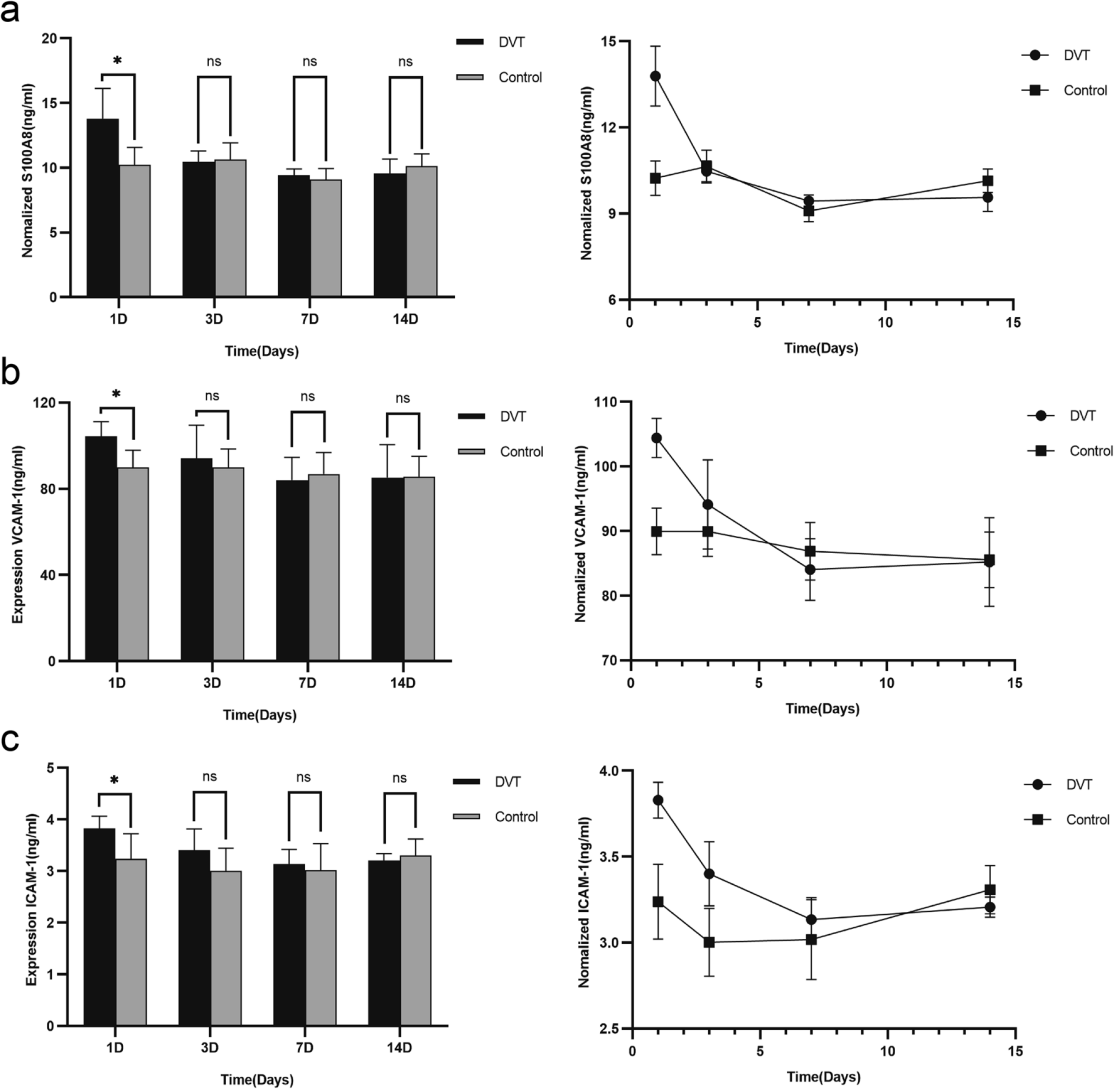
ELISA results of S100A8 (**a**), VCAM-1 (**b**), and ICAM-1 (**c**) protein levels in the plasma of DVT model rats at four time points: 1D, 3D, 7D, and 14D. ***P < 0.001, **P < 0.01, and *P < 0.05 are compared with the control group. DVT, deep venous thrombosis; ELISA, enzyme-linked immunosorbent assay; 1D, day 1; 3D, day 3; 7D, day 7; 14D, day 14.

S100A8 levels increased significantly on days 1 and 3 after DVT and decreased sharply on day 7, with no obvious changes thereafter (Fig. 6a). No differences were observed between the sham operation and normal groups at the same time points. The normalized relative expression results revealed that the expression of ICAM-1 and VCAM-1 in the other groups gradually decreased with DVT progression when compared with the 1D group; however, no significant difference in ICAM-1 was observed at two weeks (Fig. 6b and c). Therefore, plasma levels of both ICAM-1 and VCAM-1 decreased significantly with an increase in S100A8 in the first three days, and ICAM-1 returned to its original levels with a decrease in S100A8 after seven days (Supplemental table 4).

**Figure 7:**
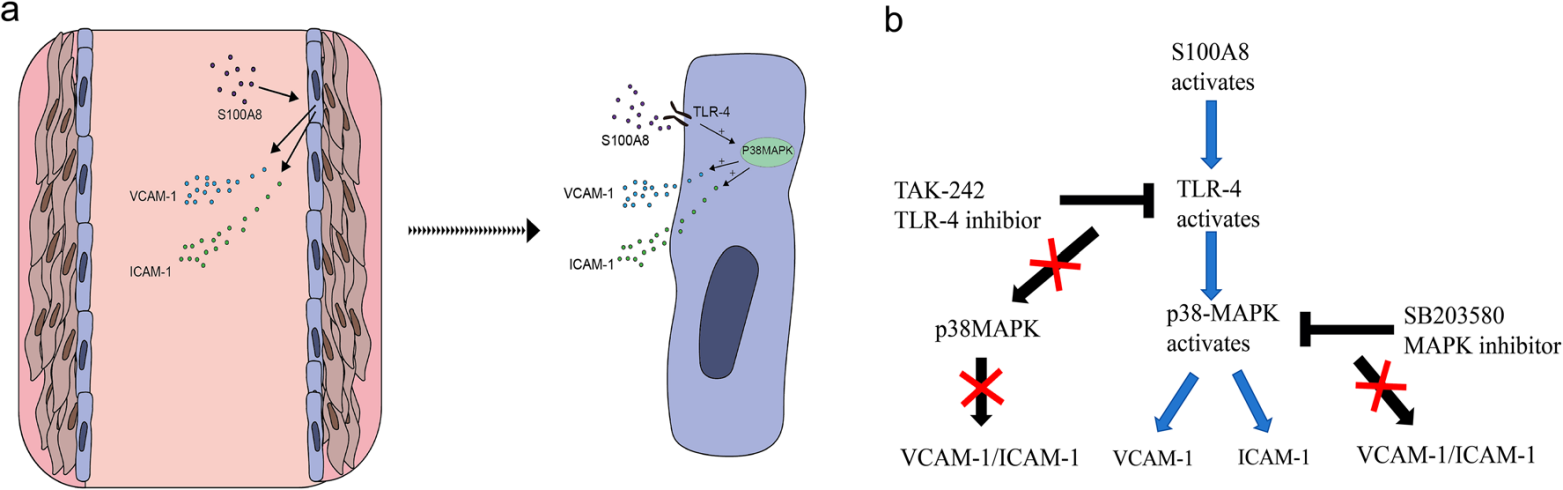
Signal transduction scheme of S100A8 in venous thrombosis. (**a**) The effect of S100A8 on venous wall endothelial cells during thrombosis. (**b**) The inhibition of TLR4 reduces p38MAPK expression, and then decreases the expression of VCAM-1 and ICAM-1. The inhibition of MAPK only decreases the expression of VCAM-1 and ICAM-1. ICAM-1, intercellular adhesion molecule 1; VCAM-1, vascular cell adhesion molecule 1; TLR4, Toll-like protein 4.

Based on the observed data, plasma S100A8 could activate p38MAPK signaling in venous wall endothelial cells and enhance the expression and secretion of VCAM-1 and ICAM-1, as well as that of S100A8 in endothelial cells via the TLR4 transmembrane protein (Fig. 7).

## Discussion

The incidence of VTE is one in 1,000 individuals worldwide and constitutes a global disease burden, with more than 50,000 new cases in the United States and 12 million new cases in China diagnosed annually^25,26^. The progression of DVT is usually relatively insidious, and the currently used DVT blood test indicator, D-dimer, is also expressed in non-thrombotic diseases, such as stroke, infection, and disseminated vascular coagulation^25,27^, indicating its poor specificity.

Therefore, we aimed to identify DVT-specific markers. We found that the expression of S100A8 in the serum of patients with DVT was significantly higher than that in the control group, as determined using a protein microarray. Moreover, we confirmed that the S100A8 protein in the blood can promote VCAM-1 expression through the TLR4/p38MAPK axis of vein wall endothelial cells. The occurrence and development of DVT altered VCAM-1 and ICAM-1 expression, S100A8 levels, and the number of inflammatory cells. These conclusions are supported by the following data: (1) S100A8 recombinant protein activated the p38MAPK signaling pathway through the TLR4 receptor on HUVECs to promote the expression of VCAM-1 and ICAM-1; (2) the levels of S100A8, VCAM-1, and ICAM-1 increased and gradually decreased in the plasma of rats and patients with acute DVT with the progression of DVT; (3) hematoxylin and eosin (H&E) staining and immunohistochemistry (IHC) revealed that intraluminal thrombus neutrophils accompanied the recanalization of DVT. The number of macrophages near the tissue gradually decreased and was replaced by a smaller number of macrophages, which is consistent with the trend of S100A8 levels in the tissue; and (4) IHC and western blotting analysis suggested that S100A8 expression on the endothelial cells of the vein wall of DVT rats abruptly decreased when compared with that in the control group on the first day, gradually returning to normal during the chronic phase. p38MAPK and VCAM-1 levels showed an opposite trend to S100A8 levels, decreasing from the first day and returning to normal levels during the chronic phase; there were no significant changes in TLR4 expression during this period.

Endothelial cells are the primary interface for communication between the thrombus and vein wall and between the thrombus and luminal occlusion caused by thrombus stimulation^28^. A growing number of studies have confirmed that inflammation-induced endothelial dysfunction is an important factor in DVT pathogenesis^16,29,30^. S100A8/S100A9 reportedly promotes the proliferation and angiogenesis of endothelial cells after activation, and the HUVEC barrier is drastically destroyed after the induction of homologous S100A8 dimers^31–33^. Activated endothelial cells express VCAM-1 and ICAM-1 to mediate neutrophil adhesion to endothelial cells and promote thrombus formation^23,34^. Our study revealed that HUVECs treated with recombinant S100A8 protein showed increased expression levels of p38MAPK, VCAM-1, and ICAM-1 when compared with the control group, and pretreatment with p38MAPK inhibitor and TLR4 inhibitor significantly inhibited VCAM-1 expression and ICAM-1 upregulation. This is consistent with the results reported by Wang et al., who reported that S100A8 can stimulate HUVECs to alter their permeability via the TLR4/p38MAPK axis^32^.

In the current study, we established the inferior vena cava (IVC) stenosis-induced DVT model in Sprague Dawley rats to simulate clinical thrombotic diseases. This model involves the partial stagnation of blood via the incomplete blockade of the inferior veins of the two kidneys^35^.Notably, a large number of studies have confirmed that this method of model construction closely approximates the clinical pathway of incomplete thrombosis due to hemodynamic changes^35–37^. Next, based on the available literature, our study evaluated the levels of S00A8, VCAM-1, and ICAM-1 protein by dividing patients in the DVT group into three time periods based on clinical symptoms and time of onset, namely, acute phase (< 15 days), subacute phase (15–30 days), and chronic phase (> 30 days)^38–43^. Additionally, Sprague Dawley rats were assigned to distinct groups according to different time periods to explore the changes in thrombus organization and protein levels in the three time periods.

Ehlermann et al. demonstrated that the heterodimer S100A8/S100A9 enhances the secretion of VCAM-1 and ICAM-1 in HUVECs in a dose-dependent manner^8^. Therefore, blood S100A8 can secrete VCAM-1 and ICAM-1 protein into the bloodstream by stimulating the secretion of VCAM-1 and ICAM-1 protein into the endothelial cells of venous walls. This was confirmed by the significantly higher plasma levels of VCAM-1 and ICAM-1 in DVT rats and patients with DVT than in those in controls within 15 days. Furthermore, plasma levels of VCAM-1 and ICAM-1 were positively correlated with S100A8 levels. All three proteins showed a downward trend over time, and the three proteins were observed to be positively correlated. Elevated levels of VCAM-1, ICAM-1, and S100A8 protein during the acute phase of DVT are closely related to endothelial dysfunction, considering that the prothrombotic state may involve the activation of endothelial cells and aggravate thrombus formation^3^. Moreover, we found that S100A8 and p38MAPK protein in the venous wall endothelial cells of DVT rats showed an opposite trend and were positively correlated with VCAM-1.

Recent studies have shown that neutrophil necrosis and platelet activation during NETosis increase S100A8/S100A9 expression in the inflammatory milieu, and these cells and protein levels are further enriched in the inflammatory milieu. Free S100A8/S100A9 protein released by neutrophils^31,44^ can affect platelet aggregation, promoting DVT^31,45^. This is similar to the difference in serum S100A8 levels in patients with DVT, as determined by enzyme-linked immunosorbent assay (ELISA). Wang et al. constructed a DVT mouse model by knocking out S100A9 in combination with partial ligation of the IVC. The authors found that S100A9 secreted by neutrophils and platelets directly regulated the formation of venous thrombosis. Based on these reports, we hypothesized that elevated S100A8 protein levels in the blood may promote DVT^18^. Therefore, we established a DVT rat model and examined the plasma of patients with DVT, revealing that levels of S100A8, VCAM-1, and ICAM-1 in both rat and human plasma were significantly increased in the early stage, gradually returning to normal levels in the late phase, as determined using ELISA. In addition, H&E staining showed that neutrophils near the thrombus tissue of rats with DVT in the acute phase were greatly enriched following DVT formation and gradually decreased with recanalization in the subacute phase. This phenomenon was positively correlated with the plasma S100A8, VCAM-1, and ICAM-1 levels. In addition, IHC identified a negative correlation between the number of wall macrophages and S100A8 levels. Colicchia et al. found that increased S100A8/S100A9 expression in the plasma of patients with COVID-19 could promote inflammation and trigger the formation of procoagulant platelets to support thrombus formation. Staining of lung autopsy specimens revealed that this complex protein was mainly deposited in neutrophils and blood vessel walls^31^, similar to the results of the current study. The increase in plasma S100A8 levels during the early stage of DVT is closely related to the number of neutrophils, which simultaneously verified that S100A8 mainly originated from neutrophils of myeloid cells; however, the specific source of S100A8, as well as how the increased S100A8 level during early-stage DVT specifically affects thrombosis, need to be further explored. However, TLR4 protein levels in rat IVC wall endothelial cells did not increase substantially during DVT development when compared with controls, similar to the results of the cellular experiments in the current study.

TLR4 is a transmembrane protein that acts as an S100A8-specific endogenous ligand and, when activated, induces diverse intracellular signal transduction pathways^19–21^. However, our results revealed that TLR4 protein levels were not significantly elevated in S100A8-stimulated endothelial cells. Inhibition of TLR4 significantly suppressed the downstream p38MAPK signaling pathway, as well as VCAM-1 and ICAM-1 proteins, suggesting that S100A8 can activate p38MAPK signaling molecules by partially binding to TLR4. This finding could be attributed to the impact of S100A8 on venous endothelial cells, which is mediated by binding to the receptor for late glycosylation end products or other receptors, leading to changes in intracellular signaling pathways, metabolic status, environmental conditions, and other factors that affect transmembrane protein (TLR4) function and quantity^46–48^. Therefore, the precise mechanism through which S100A8 protein binds to the receptor and activates the p38MAPK pathway needs to be further explored.

In summary, plasma S100A8 activates p38MAPK signaling molecules via TLR4 protein and promotes increased expression and secretion of VCAM-1 and ICAM-1 in endothelial cells. The S100A8 protein is substantially increased in the acute phase of DVT,and the number of neutrophils is closely related to the occurrence and development of DVT. Our study provides a novel basis for the early diagnosis and screening of DVT and a novel direction for the in-depth study of DVT formation mechanism.

### Limitations

Nevertheless, the limitations of this study need to be addressed. First, we did not evaluate the effect of S100A8 binding to other receptors (receptors for advanced glycation end products) to stimulate HUVECs. Second, we did not clarify the specific source of S100A8 in plasma. S100A8 and S100A9 are mainly derived from immune cells, such as neutrophils, macrophages, and monocytes. Third, we did not examine the effects of different doses of S100A8 on DVT in rats, and the effects of S100A8 on DVT after inhibition of TLR4 or p38MAPK were not investigated. In future investigations, additional experimental methods (immunoassays and flow cytometry) need to be employed to improve and clarify the mechanism of the S100A8 protein in the acute phase of DVT.

## Materials and methods

### Patients

Herein, 40 patients with newly diagnosed lower extremity DVT and 40 controls without lower extremity DVT were included based on Doppler ultrasound and D-dimer testing. The baseline information and clinical characteristics of the patients are shown in Table 1. Exclusion criteria for study participation were as follows: 1) active cancer; 2) other acute cardiovascular diseases; 3) autoimmune diseases; 4) pregnancy or puerperium; 5) pacemaker placement; 6) no past history of anticoagulants; 7) personal or family histories of VTE; or 8) received female hormonal therapy or other hormonal stimulants. Patient specimens were collected upon approval from the Institutional Ethics Committee of the First Affiliated Hospital of Hebei Northern University, and informed consent was obtained in accordance with the Declaration of Helsinki. All patient samples were de-identified and re-labeled after collection to maintain patient privacy.

### Human peripheral blood sampling and processing

After fasting for 12 h (or overnight fasting), the included subjects underwent forearm venipuncture for blood withdrawal into sodium heparin anticoagulant tubes, which were immediately transferred to a low-speed centrifuge at room temperature and centrifuged at 3000 rpm for 10 min. The supernatant was obtained by separating the peripheral blood samples over a period of 4 h. The supernatant was extracted from the peripheral blood samples and refrigerated at − 80°C until further analysis.

### G-series human cytokine antibody array 440

We first collected peripheral plasma samples from 10 patients with DVT and 10 patients without DVT (as controls) using a GSH-CAA-440 kit (RayBiotech, United States). The specific procedure was as follows. First, the plasma sample and slide chip were equilibrated at room temperature for 20–30 min and were subsequently dried for 2 h. After overnight incubation, the blocked slides were washed with deionized water on a Thermo Scientific Wellwash Versa chip washer. Next, 80 µl of detection antibody was added to each well, the wells were incubated on an RT shaker for 2 h, and the slides were subsequently washed as described earlier. Finally, InnoScan 300 (InnoScan 300 Microarray Scanner, Innopsys), a laser scanner, was used to scan the signal using Cy3 or a green channel (excitation frequency = 532 nm), and the obtained data were analyzed using the Bioconductor org.Hs.eg.db (https://bioconductor.org/packages/org.Hs.eg.db/).

### ELISA

Additionally, we collected plasma from 30 patients with DVT and 30 controls. The samples were stored at − 80°C, and protein analysis was performed using the Human S100A8 ELISA Kit (Shanghai Jining, 96T, China). Samples were equilibrated at room temperature 30 min prior to the assay. In brief, 10 µl of the prepared sample was added to 40 µl of standard and reacted at 37℃ for 30 min. Next, the plate was washed five times, with an interval of 30 s between each wash cycle, and dried. The enzyme labeling reagent was added, and the plates were incubated at 37℃ for 30 min; the plates then were washed and dried five times, with an interval of 30 s between each wash cycle. The color developers A and B were added, and plates were incubated at 37℃ for 10 min. Finally, the termination solution was added, and the optical density was detected within 15 min. Likewise, we detected protein levels of VCAM-1 (Shanghai Jining, 96T, China) and ICAM-1 (Shanghai Jining, 96T, China).

### In vitro* experiment*

#### Cell culture

HUVECs (ScienCell, 8000, United States) were recovered and maintained in DMEM basal medium (Thermo Fisher Scientific) containing 10% fetal bovine serum (FBS) and penicillin–streptomycin (03–031-1b; Biological Industries, China and C11995500BT; United States) and placed in an incubator at 37 °C and under 5% CO_2_ saturated humidity for 12 h. The original culture medium was removed, and the cells were digested with 0.25% EDTA trypsin (C3530-0100; VivaCell, China) for 3 min at 37 °C to induce shrinkage. After centrifugation, the cells were seeded into 2–3 culture flasks for passaging. The culture medium was changed every 1–2 days according to the cell growth status and experimental schedule.

#### Stimulation of HUVECs by S100A8 recombinant protein

For pretreatment interventions, HUVECs were divided into 6 groups: (1) S100A8 5 µg/ml; (2) S100A8 5 µg/ml + SB203580 10 µM; (3) S100A8 5 µg/ml + TAK-242 10 µM; (4) HUVECs; (5) SB203580 10 µM; and (6) TAK-242 10 µM. During the intervention, the cells were first incubated with inhibitors for 30 min, followed by the addition of exogenous S100A8 (P00431; Solarbio, China) for 6 h. The cells were collected and analyzed using western blotting.

### Western blotting

Briefly, pre-cooled RIPA lysis buffer (C1053; Applygen, China) was added at 100 mg/1 ml, and protease inhibitor (P1265; Applygen, China) was added at a ratio of 1:49 according to RIPA lysis. Cells were homogenized and lysed on ice until 95% of the cells were ruptured. Cells were then placed in an ice bath for 10 min and rotated every 30 s for 5 min. Protein concentration was determined using a bicinchoninic acid (BCA) protein quantification kit (P1511; Applygen, China). The samples were denatured at 95 °C for 5 min in 5 × SDS-PAGE Protein Sample Loading Buffer (B1012; Applygen, China), dissociated on a polyacrylamide gel, and then transferred to Polybius. The samples were blocked with 5% skim milk on a polyvinyl fluoride membrane and placed on a shaker for 1 h. The membranes were then overnight incubated with anti-S100A8 (ab180735; Abcam, United Kingdom), anti-TLR4 (13,871; Cayman, United States), anti-p38MAPK (bs-0637R; Bioss, China), anti-ICAM-1 (bs-0608R; Bioss), and anti-VCAM-1 (bs-0396R; Bioss) at 4 °C. Subsequently, the indicated goat anti-rabbit secondary antibody (C31460100C; Thermo Fisher, United States) was used to bind the primary antibody. An ECL chemiluminescence substrate (P0018S; Beyotime, China) and a chemiluminescence instrument (Clinx Science Instruments Co.Ltd., ChemiScope 6200, China) were used for illustration. Blots were subjected to densitometric analysis using image-J Ij(https://imagej.nih.gov/ij).

### In vivo* experiment*

#### Animals

A total of 60 male Sprague Dawley rats weighing 275 ± 25 g and aged 8–10 weeks old were purchased from Spefford Company (110,324,231,106,204,112, China). All rats were maintained in an SPF-grade animal room at a temperature of 24 ± 5 °C and under a 12/12 h light/dark cycle. The rats had free access to a uniform diet and drinking water during the feeding period. Rats were randomly divided into a model group (M, n = 20) and a control group (F, n = 20). Each group was further divided into four subgroups (n = 5 for each subgroup in the M group and n = 5 for each subgroup in the F group) on days 1, 3, 7, and 14. Animal experiments were based on ethical considerations and integrity-based assumptions. All experimental protocols were approved by the Institutional Animal Ethics Committee of the First Affiliated Hospital of Hebei Northern University and followed the guidelines of the Animal Experiments Control and Supervision Committee.Furthermore, this study adheres to the recommendations of the ARRIVE guidelines (https://arriveguidelines.org) for the reporting of animal experiments.

### Construction of rat IVC thrombosis stenosis model

DVT was induced by the incomplete ligation of both infrarenal veins, leading to partial blood stasis, as described previously, with slight modifications^35^. The rats were fasted for 8 h prior to surgery, with free access to water. Sprague Dawley rats weighing 275 ± 25 g were placed in the isoflurane induction chamber in a quiet environment. After the induction of anesthesia, rats were taken out from the chamber, administered 1% pentobarbital at a rate of 100 mg/kg intramuscularly, and then placed in the observation room for 3–5 min. After ensuring that the rats were fully anesthetized, an electric razor was used to shave the abdominal region, from the subacromial region to the inguinal region, and the lower limbs of the rats.A 2 cm incision was made along the midline of the abdomen, and a moist cotton ball was used to move the internal organs to the periphery of the abdominal cavity to expose the IVC, abdominal aorta, and left and right iliolumbar veins. A 30G needle was placed parallel to the IVC and distal to the left renal vein, and the stenosis was created by simultaneous ligation with a 7–0 gauge wire; the needle was then removed^36^. Next, 7–0 sutures were used to ligate the bilateral iliolumbar veins, dorsal reflux vein of the IVC, and visible reflux of the IVC collaterals. The internal organs were returned after the operation, and the abdominal muscles and skin were sutured. This mechanism of DVT formation is similar to that of clinical incomplete thrombosis caused by hemodynamic changes^37^. The sham operation group was performed similarly to the induction of the IVC stenosis model, except for the vein ligation^49^. Subsequently, the rats were maintained in an SPF-level animal room. IVC blood vessels, thrombus tissue, and plasma samples were collected after day 1 (1D), day 3 (3D), day 7 (7D), and day 14 (14D)^50^.

### Evaluation of animal model construction and sample collection

To determine the degree of stenosis at the ligation site, we measured blood flow velocities in the femoral veins of both lower limbs of rats using laser Doppler flowmetry before and within 3–5 min after surgery and before sampling (Supplemental file 5) at the four preset time points (1D, 3D, 7D, and 14D after modeling. The rats were anesthetized using the above-described method. The abdomen was opened, and the internal organs and other tissues were dissociated. Rat blood was collected through the portal vein. The mixture was centrifuged at 3000 rpm for 10 min at room temperature. The plasma was aliquoted and frozen in a − 80 °C refrigerator until analysis. Next, the tissue at the ligation site was cautiously freed to expose the IVC maximally, and the vessel and thrombus tissue were excised using tissue scissors. The lengths of the IVC and thrombus were measured using a Vernier caliper (Supplemental file 6) , and the samples were quickly frozen in liquid nitrogen.

### H&E staining

Whole thrombus and vascular (IVC) tissues were stripped and fixed in 4% formaldehyde. Subsequently, specimens were transferred to gradient ethanol solutions, sequentially immersed for dehydration, followed by immersion in xylene I and xylene II for 30 min each for dewaxing; finally, the tissues were placed in paraffin I, paraffin II, and paraffin III for 1 h each (wax dipping). Sections were cut into 5 µm thickness, transferred onto glass microscope slides, and placed in the oven for 30 min at 60℃. The sections were then subjected to H&E staining for routine histomorphometric examination. Briefly, slides were immersed in xylene I and II each for 10 min for dewaxing; this was followed by immersion in gradient alcohol concentrations and rehydration in water for 5 min. The slides were then rinsed under running water for 10 min, followed by hematoxylin staining for 3 min and slow rinsing under running water for 5 min. Upon color development to blue, the sections were faded using hydrochloric acid–ethanol solution, and the color changed to light red in 3–5 s. The slides were then rinsed under running water until the color was restored to blue. Eosin staining was performed for 3 min, and the slides were rinsed under running water for 3 min for ethanol removal. For dewaxing, the slides were immersed in xylene I and II for 5 min. Ultimately, the slides were sealed with neutral gum and preserved.

### IHC of IVC

Tissue fixation, dehydration, wax dipping, embedding, sectioning, and baking were performed as described in the H&E staining protocol. Then, the slides were deparaffinized with xylene and ethanol, followed by antigen repair, cooling, and washing. After blocking with endogenous peroxidase blocker (PV-6001; Zsbio, China), the slides were blocked with closed goat serum (SL038; Solarbio, China) for 30 min; the blocking solution was removed, and the sections were incubated with prepared CD68 polyclonal antibody (GB113109-100; Servicebio, China) as the primary antibody at 4℃. The sections were incubated with prepared CD68 polyclonal antibody (GB113109-100; Servicebio) as the primary antibody at 4℃ for 50 min and then incubated with horseradish peroxidase-labeled goat anti-rabbit antibody (C31460100C; Thermo Fisher, USA) as the secondary antibody for 50 min. After washing and drying the slides, DAB solution was added for color development; the reaction was terminated when a brownish-yellow color was achieved. The sections were first stained with hematoxylin, followed by the addition of hematoxylin differentiation solution, and the blue color was restored using a hematoxylin reblue solution; the slides were rinsed with tap water at the end of each step. The sections were sealed and photographed to record the mononuclear macrophages. The same immunostaining method was used to detect S100A8 (CD20093; Chundubii, China).

### Western blotting

Briefly, frozen tissue samples were removed from the liquid nitrogen tank and cut into blocks on ice. Pre-cooled RIPA lysis buffer (C1053; Applygen) was added at 100 mg/1 ml, and protease inhibitor (P1265; Applygen) was added at a ratio of 1:49, according to RIPA lysis. Tissue lysates were centrifuged at 12,000 × g for 10 min at 4°C, and the supernatant was transferred to a new centrifuge tube. Protein concentration was determined by the BCA protein quantification kit (P1511; Applygen). The remaining steps were performed as described previously (In vitro western blotting experiments).

### ELISA

Samples were equilibrated at room temperature 30 min prior to the assay and assayed using the Rat S100A8 ELISA kit (CD20093; Chundubio, China) equilibrated for 1 h. In brief, 10 µl of the prepared sample was added to 40 µl of standard and reacted at 37℃ for 30 min. Next, the plate was washed five times, with an interval of 30 s between each wash cycle, and dried. The enzyme labeling reagent was added at 37℃ for 30 min; the plates were then washed and dried five times, with an interval of 30 s between each wash cycle. The color developers A and B were added, and plates were incubated at 37℃ for 10 min. Finally, the termination solution was added, and the optical density was detected within 15 min. Plasma protein levels of ICAM-1 (CD16298; Chundubio) and VCAM-1 (CD12470; Chundubio) in peripheral blood collected from Sprague Dawley rats were similarly detected and assayed as described previously.

### Statistical analyses

All experiments were repeated at least three times. Normally distributed data were compared using independent sample t-tests; the data are expressed as means ± standard deviations. Categorical variables are expressed as frequencies and percentages, with differences compared using the chi-square test or fisher's exact test. One-way ANOVA was used when homoscedasticity was assumed; otherwise, the data after transformation were analyzed using the Kruskal–Wallis test. Correlation coefficients were calculated using the Spearman’s correlation coefficient. All tests were 2-sided, and statistical significance was set at *P* < 0.05. All statistical analyses were performed using SPSS for Windows version 25.0 (IBM Corp., Armonk, NY, USA,http://www.ibm.com/developerworks/spssdevcentral).Plotting with Graphpad prism 9.0.0(http://www.graphpad.com/updates/prism-900-release-notes).

## Ethical approval and consent to participant

The experimental operation was approved by the Institutional Ethics Committee of the First Affiliated Hospital of Hebei North University (Approval No.K2020240). The animal experiments were in accordance with the statement on animal Ethics Review of Hebei North University (Approval No.HBNU2023012006). Furthermore, this study adheres to the recommendations of the ARRIVE guidelines (https://arriveguidelines.org) for the reporting of animal experiments. All animal experiments performed in this study were approved by the relevant ethics committees, and all experimental procedures comply with local laws and ethical standards.

### Supplementary Information

Supplementary Information 1.

Supplementary Information 2.

Supplementary Information 3.

Supplementary Information 4.

Supplementary Information 5.

Supplementary Information 6.

Supplementary Information 7.

Supplementary Information 8.

Supplementary Information 9.

Supplementary Information 10.

## Data Availability

All data generated or analyzed during this study are included in this published article [and its supplementary information files].
