# Supplementary material for: Preliminary clinical analysis and pathway study of S100A8 as a biomarker for the diagnosis of acute deep vein thrombosis
Source: Sci Rep. 2024 Jun 10;14:13298. doi: 10.1038/s41598-024-61728-6 (PMC11164926; doi:10.1038/s41598-024-61728-6)
Supplement: Supplementary file 1 — Supplementary Information 1. [file 41598_2024_61728_MOESM1_ESM.docx]

First of all, according to the first day, the third day, the seventh day and the fourteenth day,Western blot were used to detect the five protein of TLR-4, ICAM-1, VCAM-1, p38 MAPK and S100A8 in the inferior vena cava wall of the DVT group and the control group. The original map was repeated 3 times in each group.


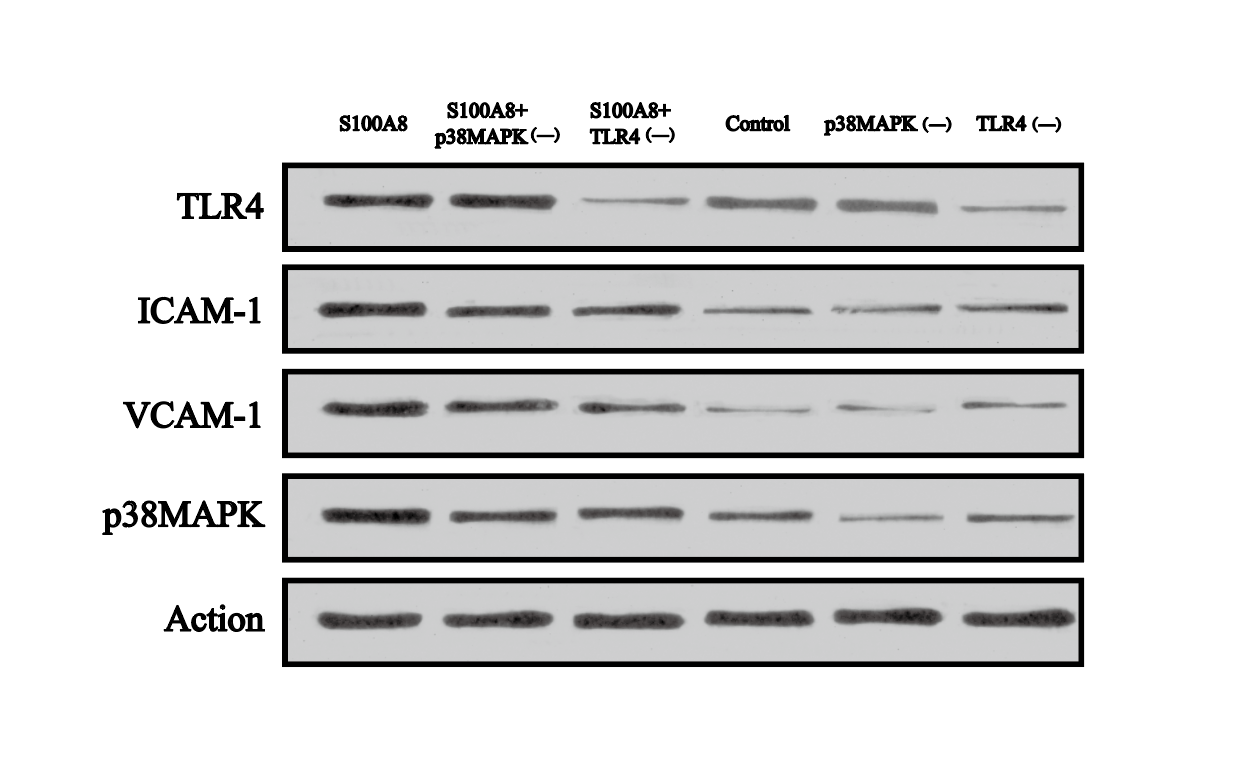


Figure 3 in the paper.(Western blot of cell)

(a)(b)(c) is origin figure of TLR4 which was repeated 3 times(Western blot of cell):


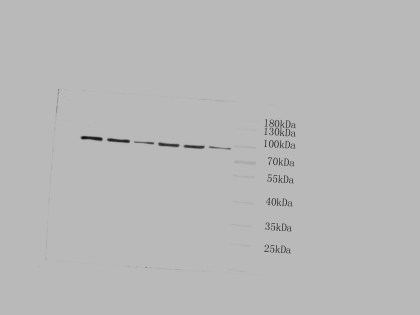

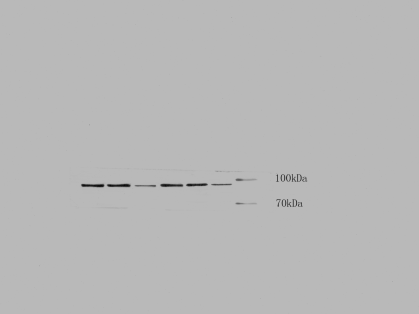

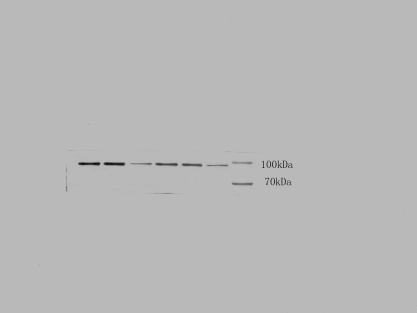


a.TLR-4 (1) b.TLR-4 (2) c.TLR-4 (3)

(d)(e)(f) is origin figure of ICAM-1 which was repeated 3 times(Western blot of cell):


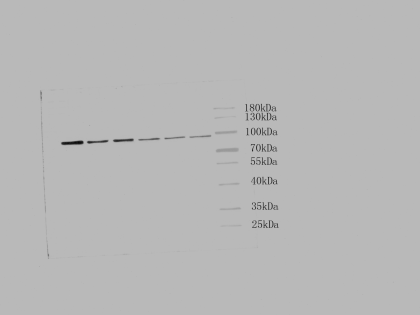

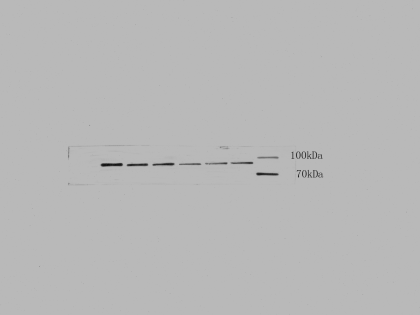

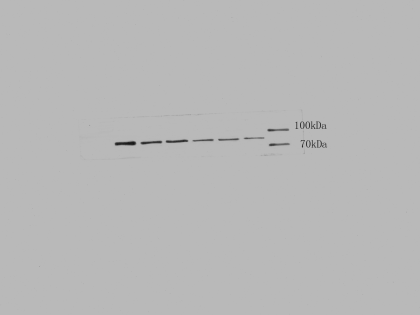


d.ICAM-1 (1) e.ICAM-1 (2) f.ICAM-1 (3)

(g)(h)(i) is origin figure of VCAM-1 which was repeated 3 times(Western blot of cell ):


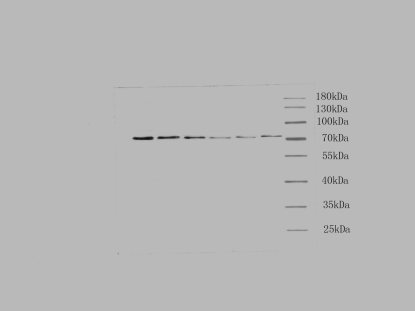

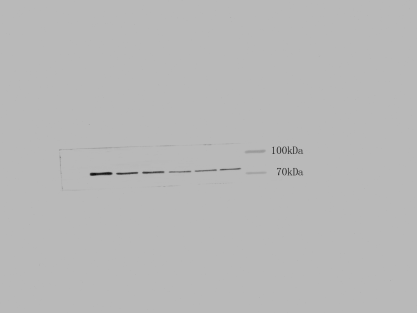

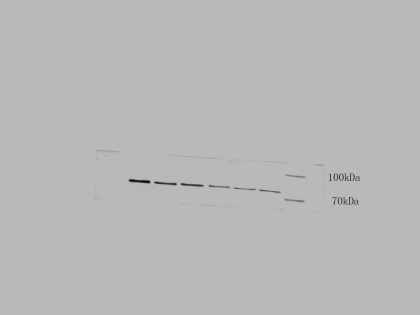


g.VCAM-1 (1) h.VCAM-1 (2) i.VCAM-1 (3)

(j)(k)(l) is origin figure of P38MAPK which was repeated 3 times(Western blot of cell):


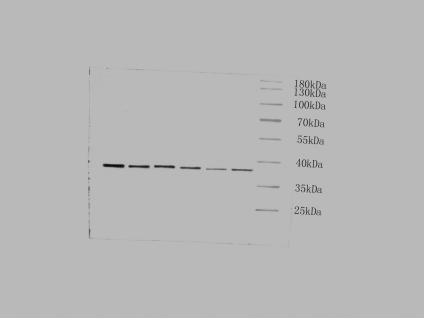

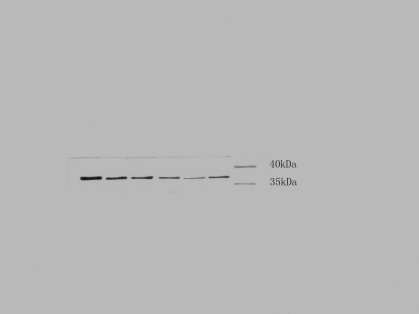

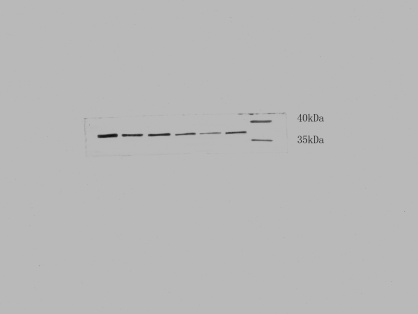


j.p38MAPK (1) k.p38MAPK (2) l.p38MAPK (3)

(m)(n)(o) is origin figure of Actin which was repeated 3 times(Western blot of cell):


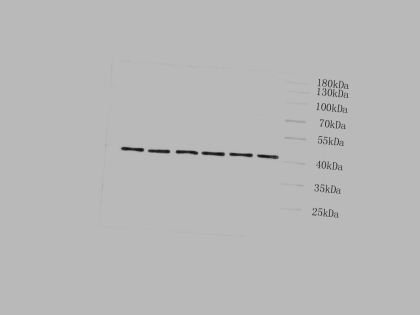

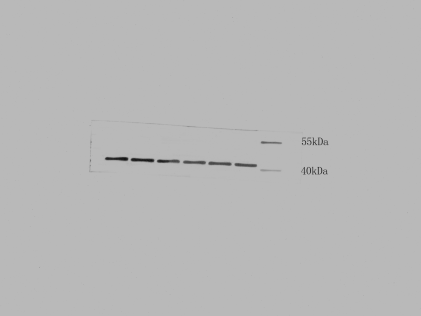

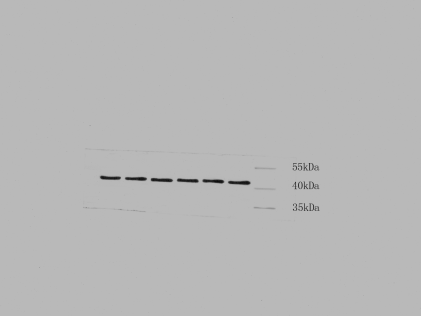


m.actin (1) n.actin (2) o.actin (3)

Then Western blot was used to detect five protein of TLR-4, VCAM-1, p38 MAPK and S100A8 in the inferior vena cava wall of rats in the DVT group and the control group according to the first day, the third day, the seventh day and the fourteenth day. The original map was repeated at least 3 times in each group.


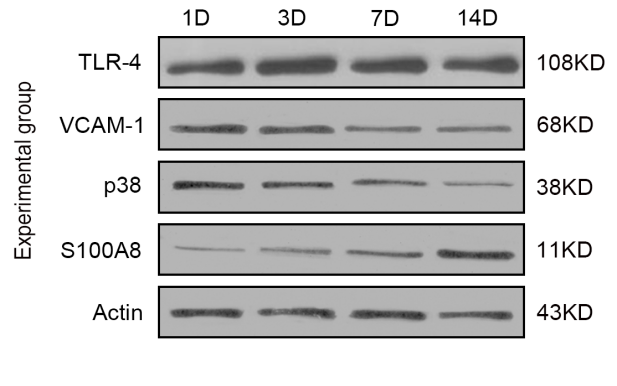

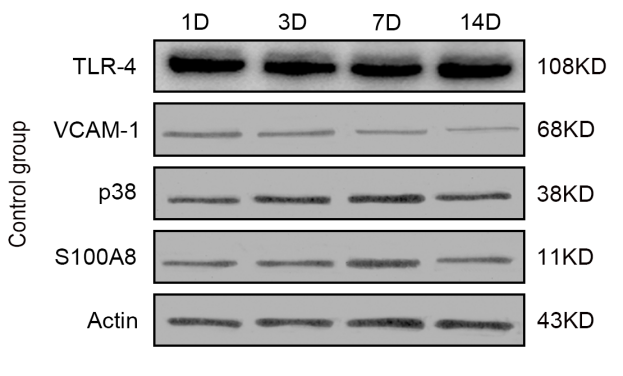


This figure comes from Figure 5A the manuscript.(Western blot of SD rat)

B(1-3) is origin image of TLR-4 with corresponding actin which was repeated 3 times.(Western blot of SD rat)


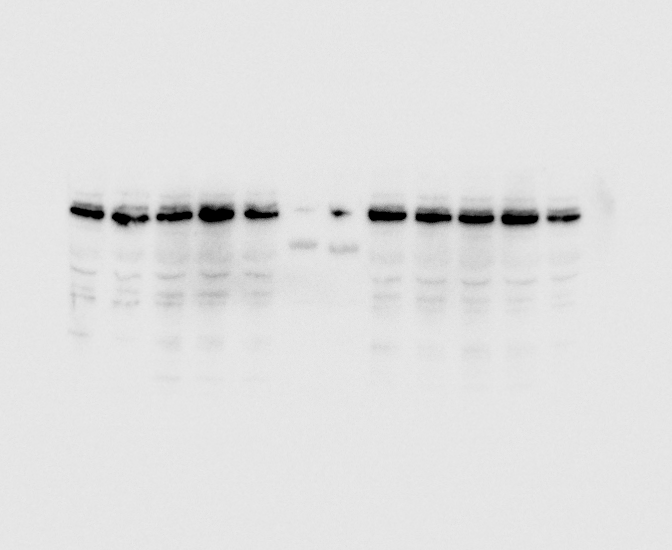

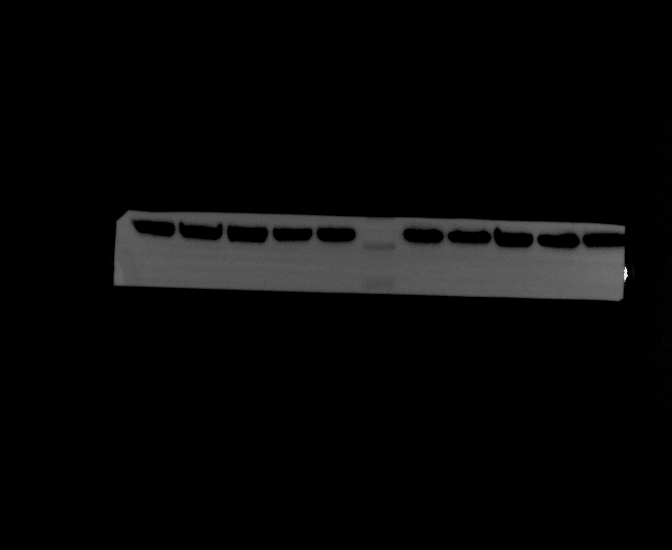


B(1) TLR-4 B(1) actin


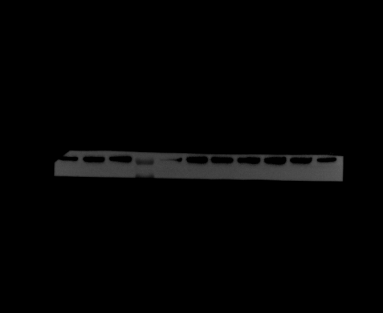

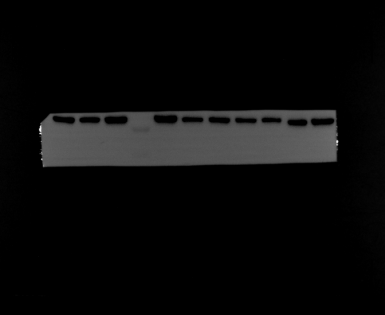


B(2) TLR-4 B(2) actin


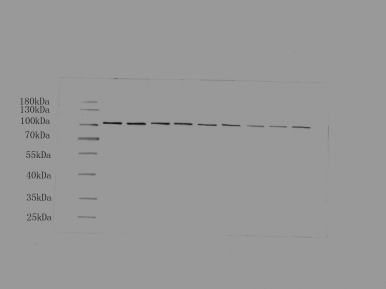

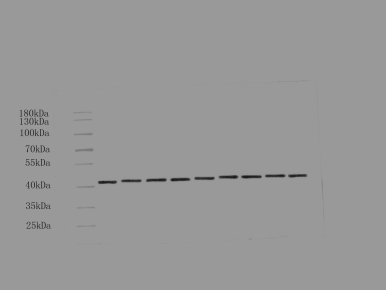


B(3) TLR-4 B(3) actin

C(1-3) is origin image of VCAM-1 with corresponding actin which was repeated 3 times.(Western blot of SD rat)


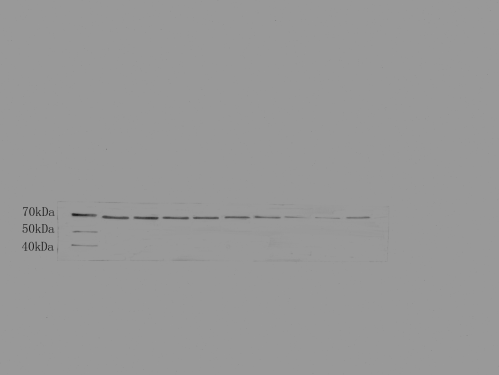

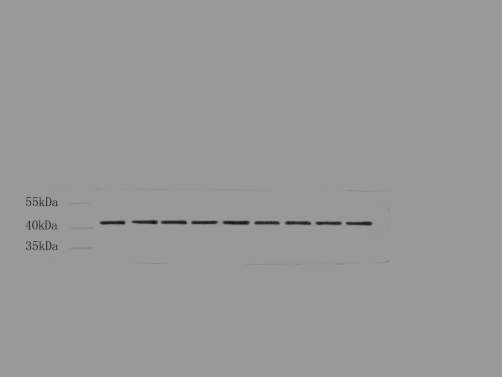


C(1) VCAM-1 C(1) actin


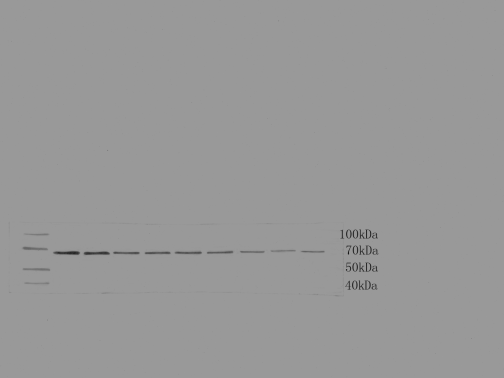

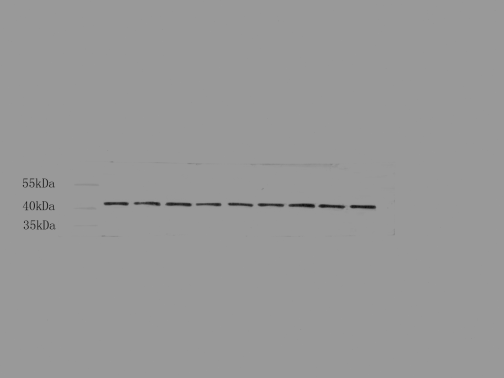


C(2) VCAM-1 C(2) actin


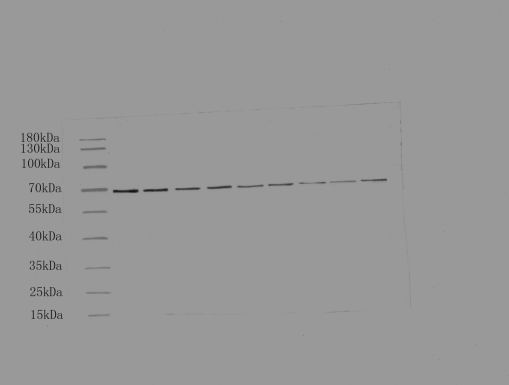

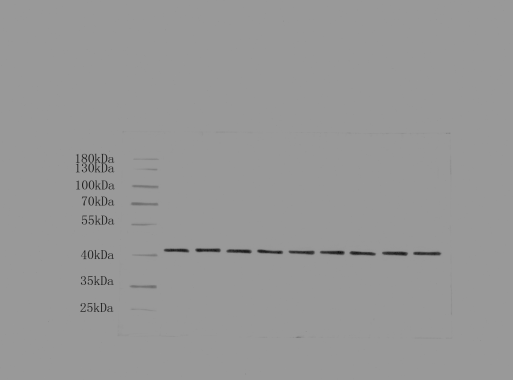


C(3) VCAM-1 C(3) actin

D(1-3) is origin image of p38MAPK with corresponding actin which was repeated 3 times.(Western blot of SD rat)


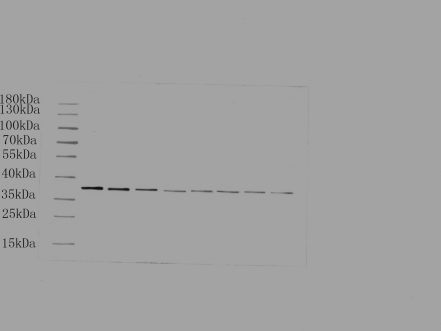

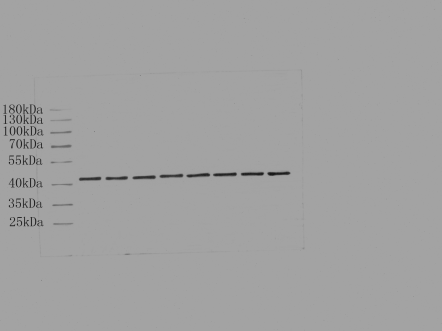


D(1) P38MAPK D(1) actin


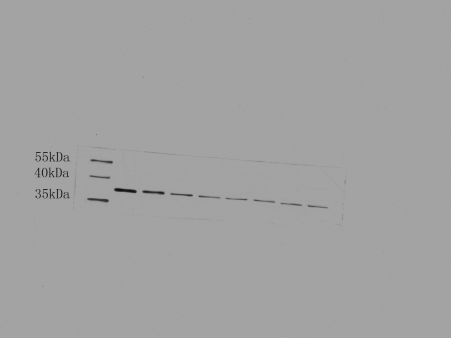

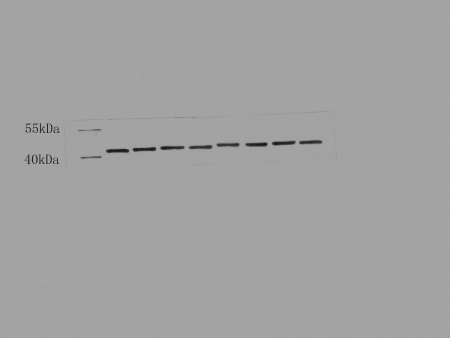


D(2) P38MAPK D(2) actin


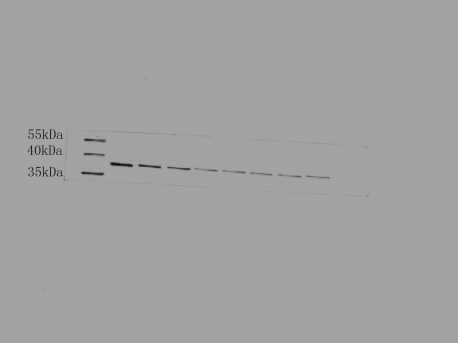

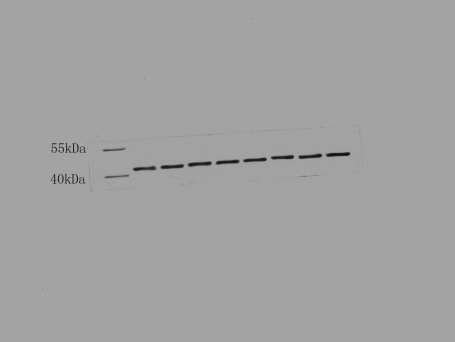


D(3) P38MAPK D(3) actin

E(1-3) is origin image of S100A8 with corresponding actin which was repeated 3 times.(Western blot of SD rat)


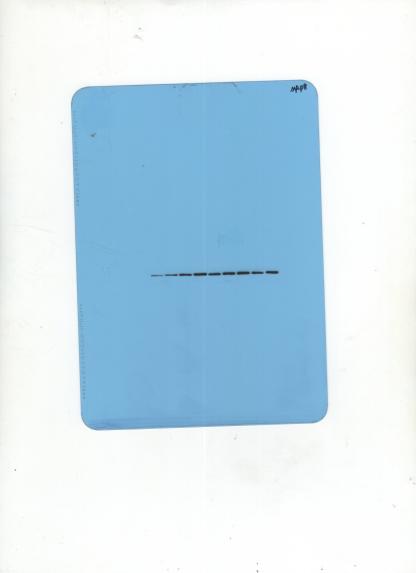

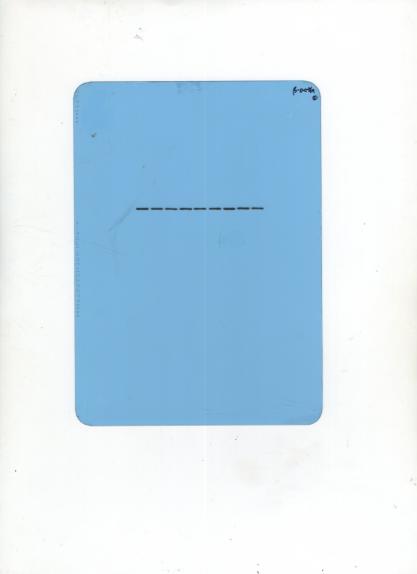


E(1) S100A8 E(1) actin


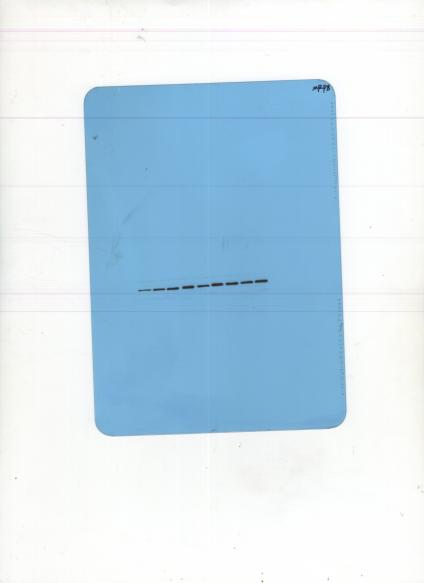

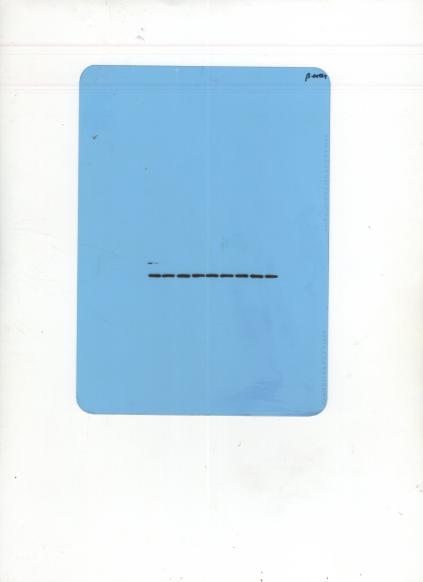


E(2) S100A8 E(2) actin


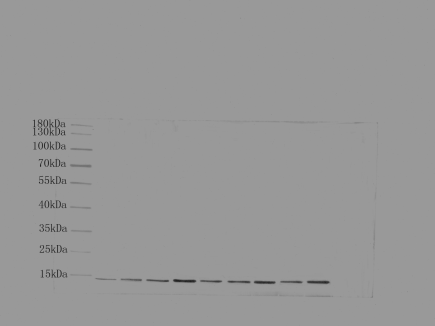

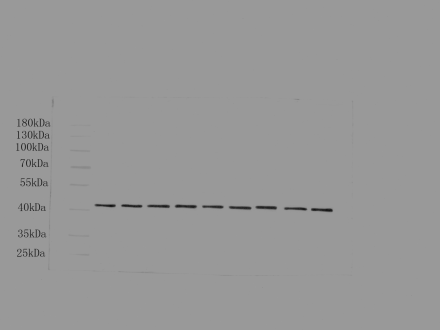


E(3) S100A8 E(3) actin
