# Supplementary material for: Preliminary clinical analysis and pathway study of S100A8 as a biomarker for the diagnosis of acute deep vein thrombosis
Source: Sci Rep. 2024 Jun 10;14:13298. doi: 10.1038/s41598-024-61728-6 (PMC11164926; doi:10.1038/s41598-024-61728-6)
Supplement: Supplementary file 4 — Supplementary Information 4. [file 41598_2024_61728_MOESM4_ESM.docx]

Immunohistochemistry was used to verify the **S100A8** protein in the inferior vena cava and thrombus tissues of the DVT group and the control group at the four time points of the first day, the third day, the seventh day and the fourteenth day.

**Figure(A)(B)(C) is the tissue of** **three DVT SD rats on the first day** **respectively.**

**
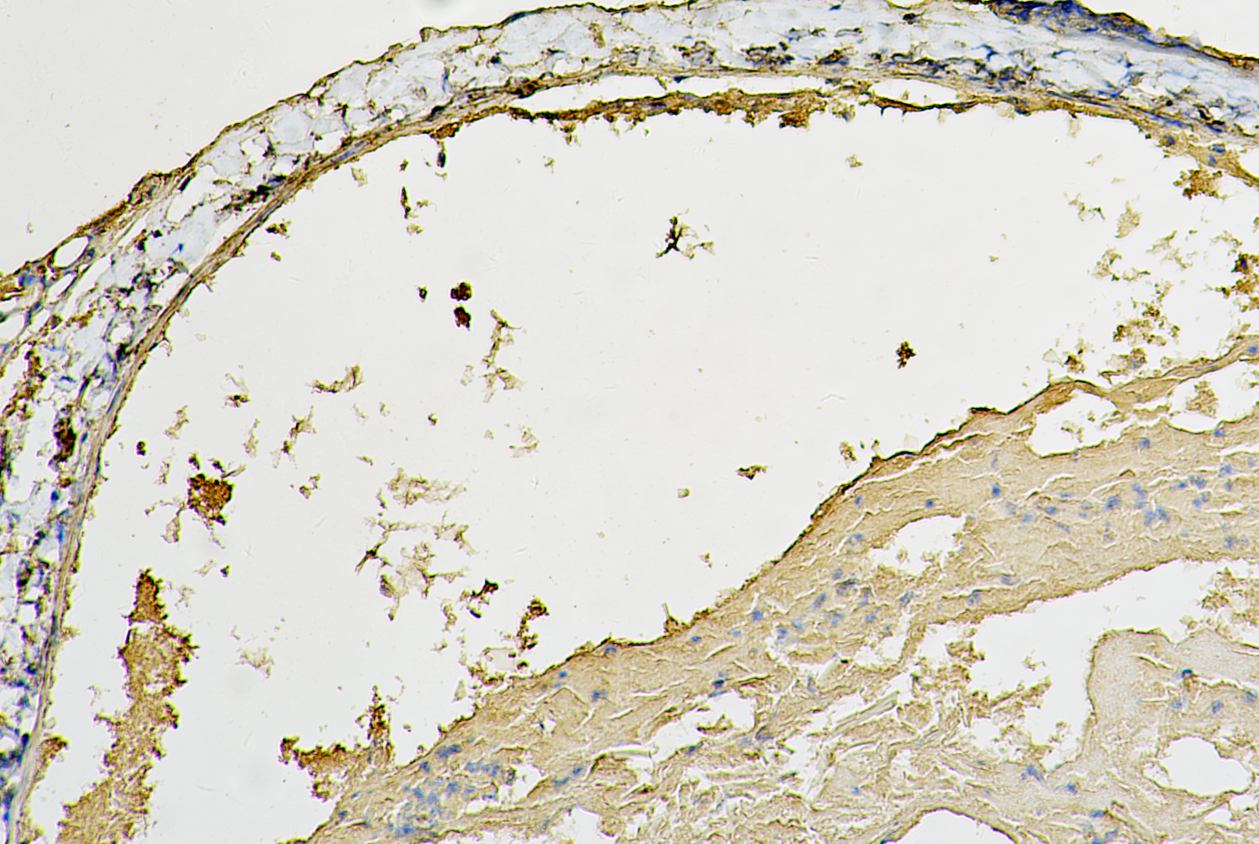
**

**Figure A.DVT-1D-1**

**
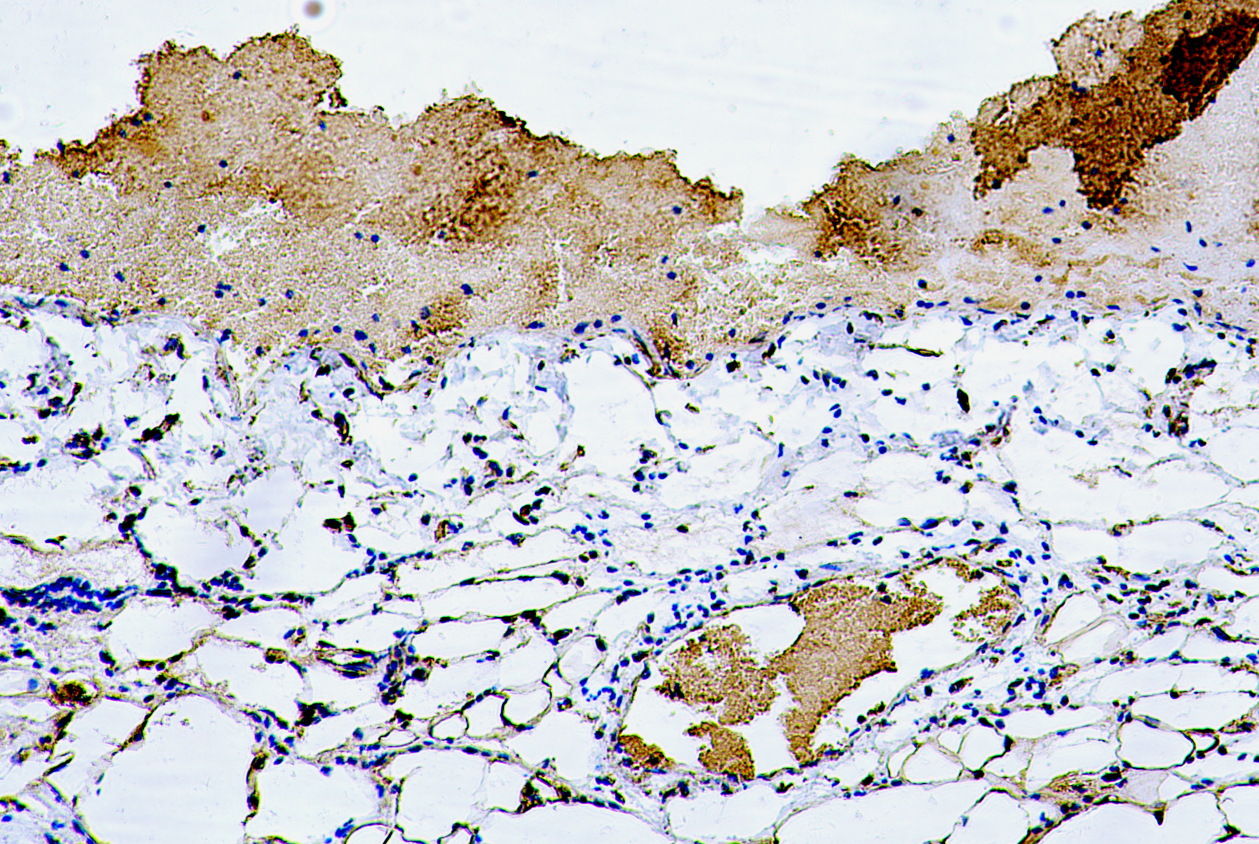
**

**Figure B.DVT-1D-2**

**
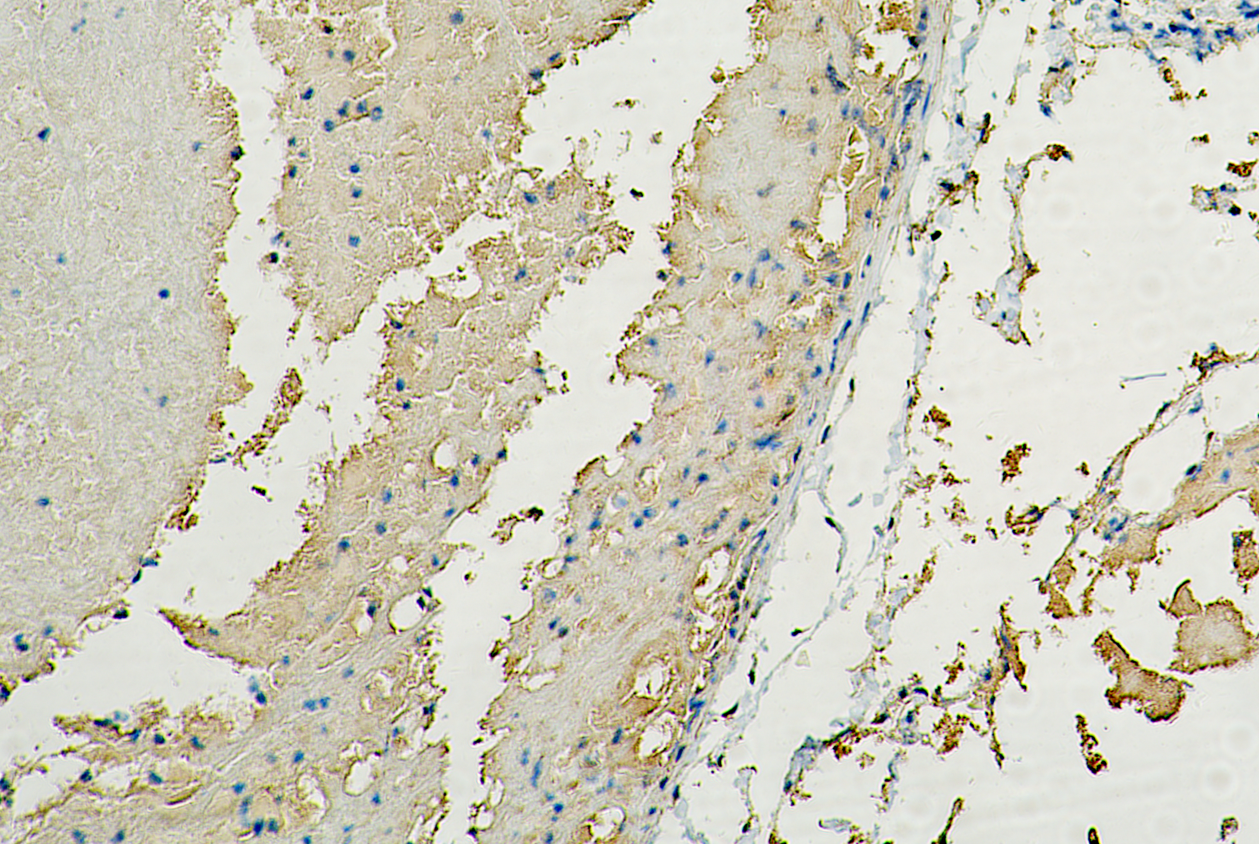
**

**Figure C.DVT-1D-3**

**Figure(D)(E)(F) is the tissue of three control SD rats on the first day** **respectively.**

**
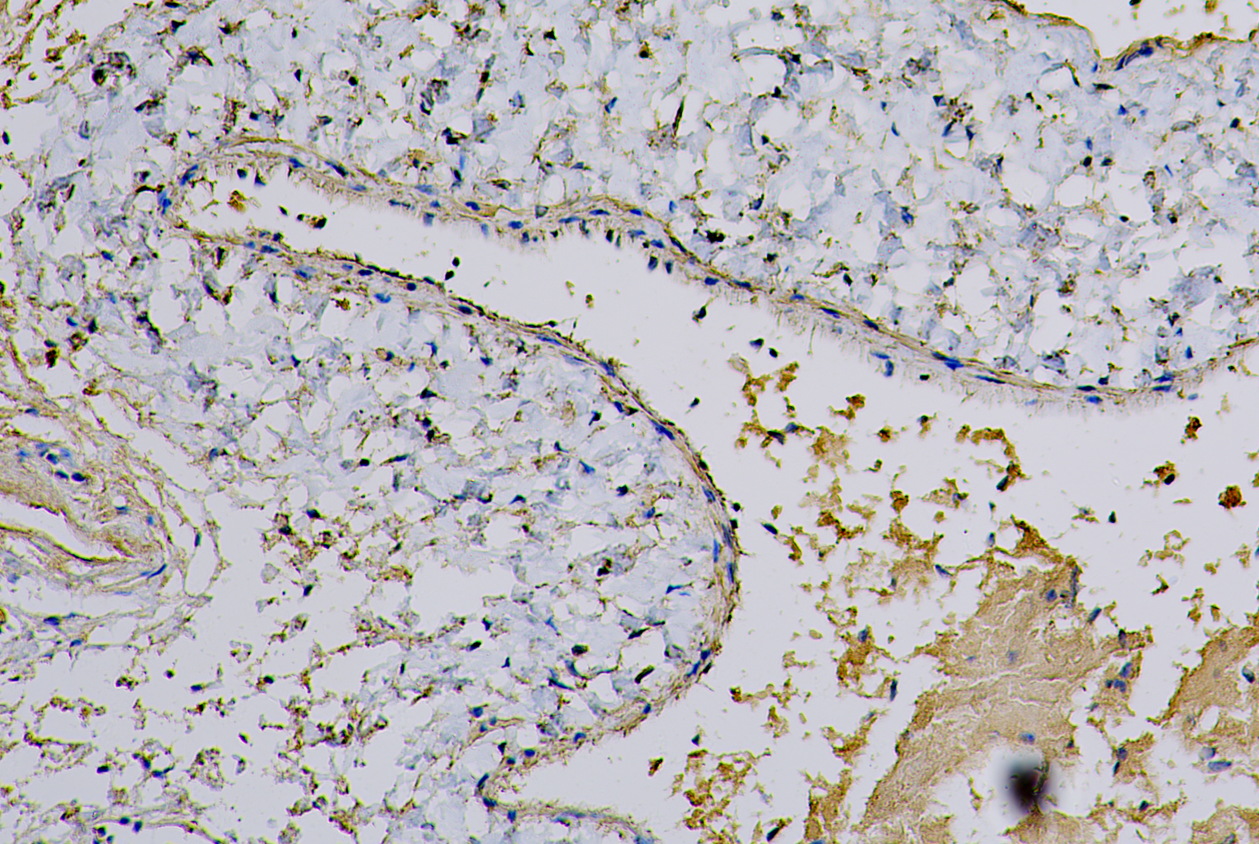
**

**Figure D.****Control-1D-1**

**
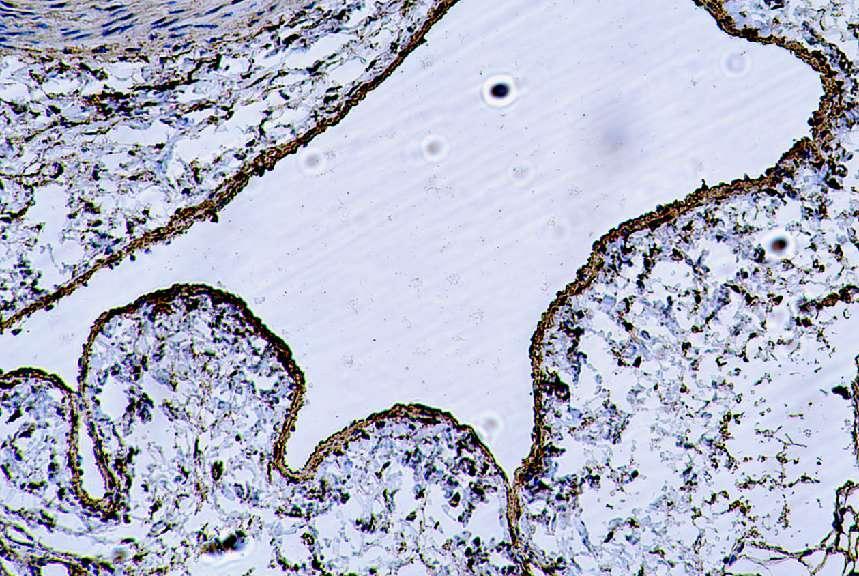
**

**Figure E.Control-1D-2**

**
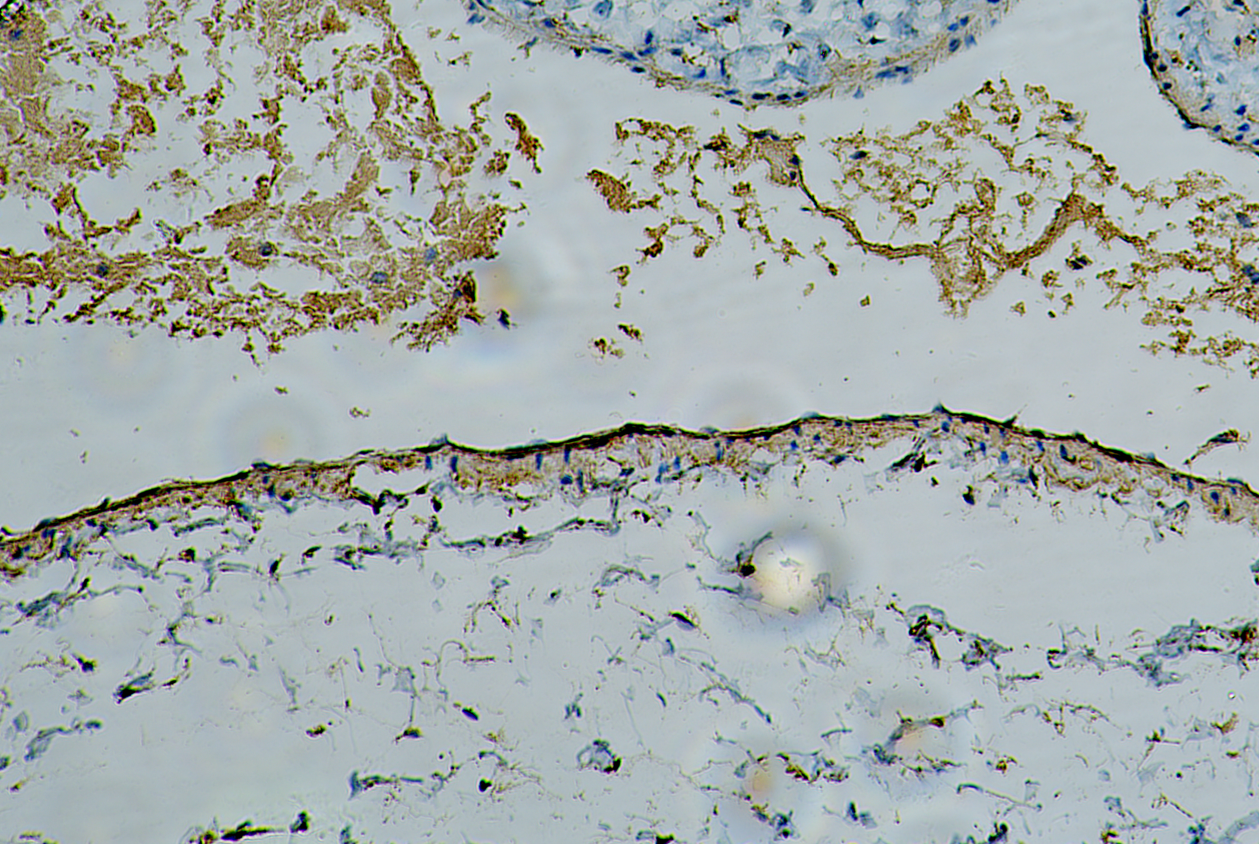
**

**Figure F.Control-1D-3**

**Figure(G)(H)(I) is the tissue of one DVT SD rats on the** **third day respectively.**

**
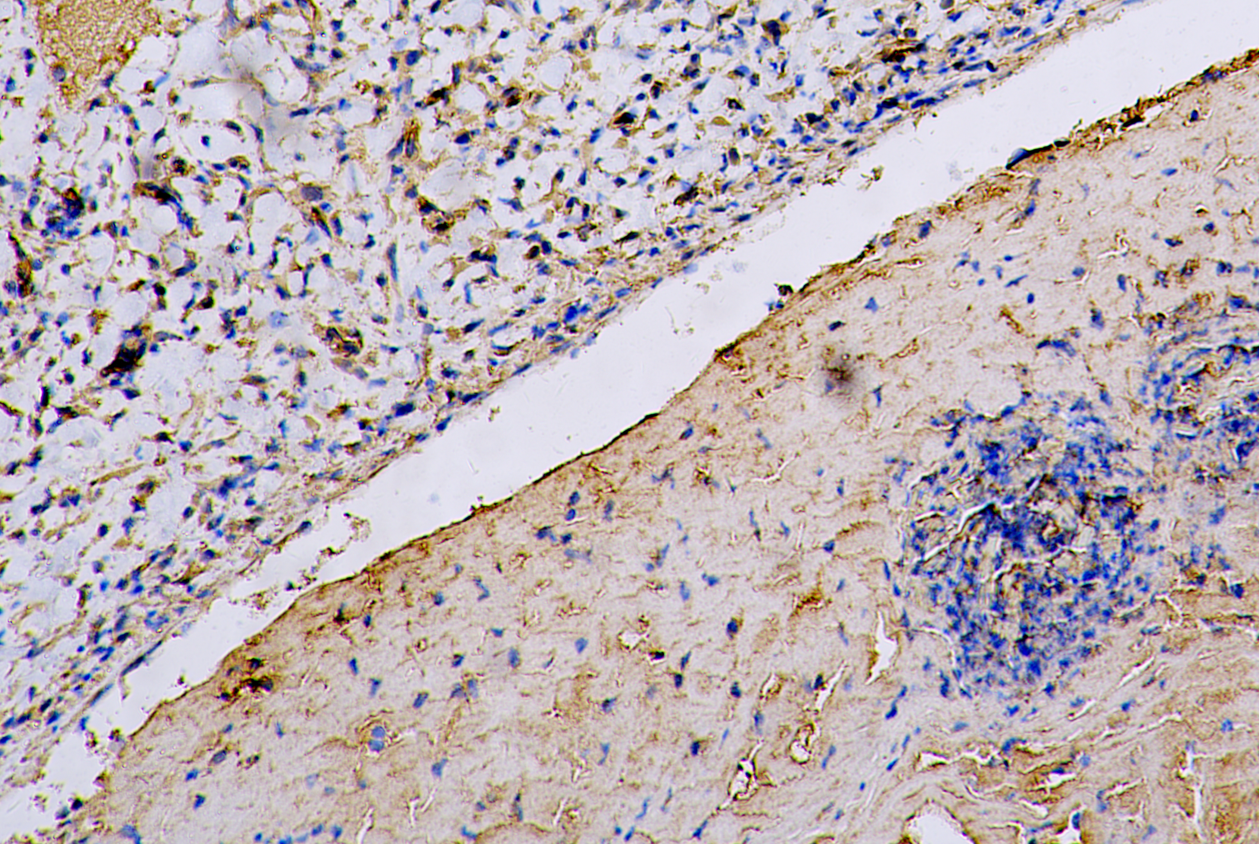
**

**Figure G.DVT-3D-1**

**
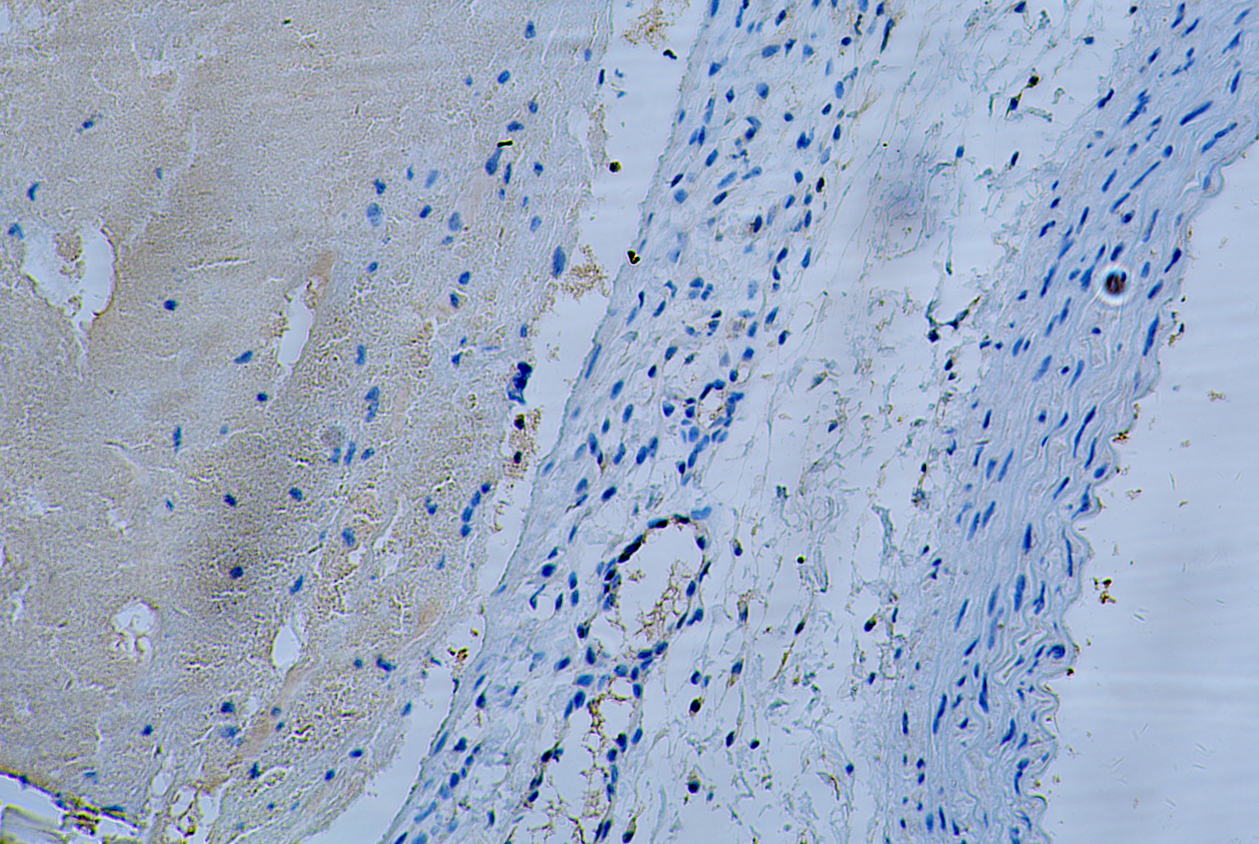
**

**Figure H.DVT-3D-2**

**
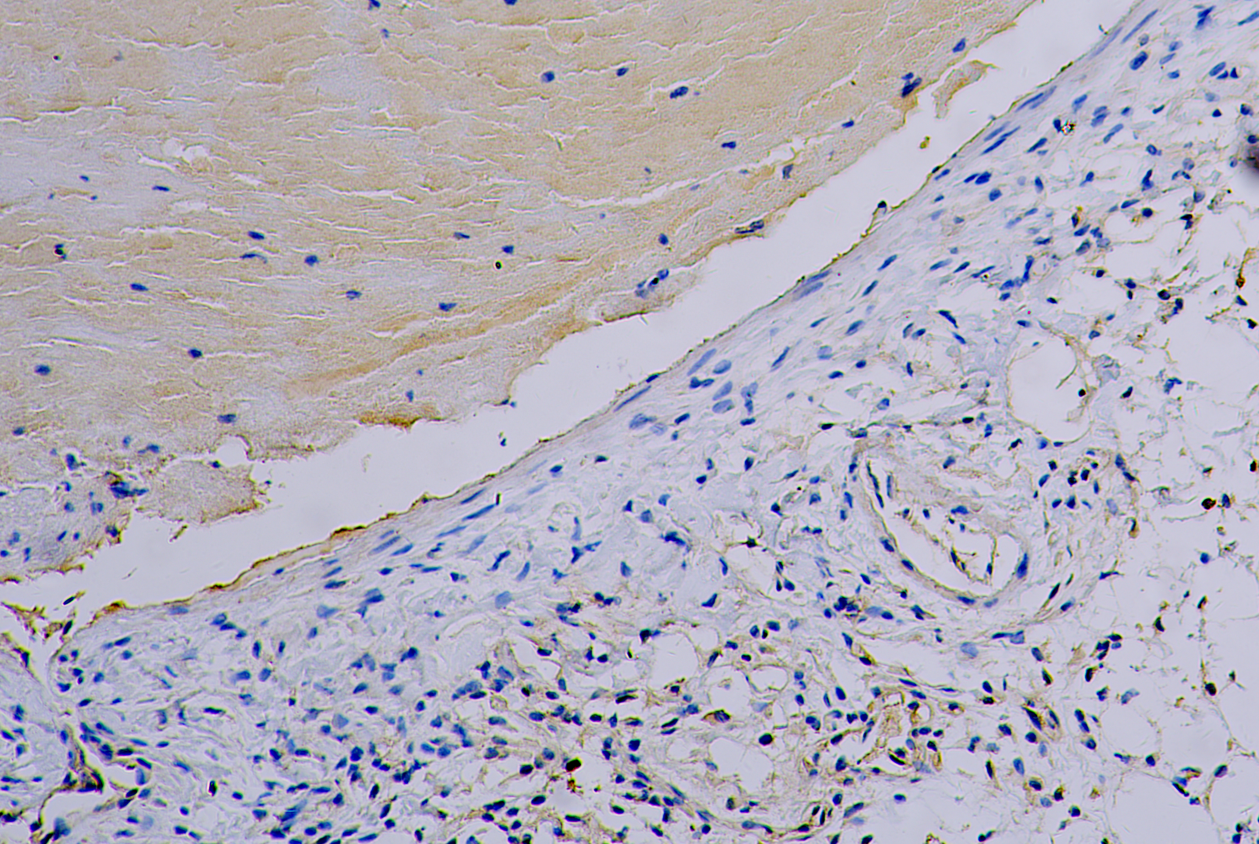
**

**Figure I.DVT-3D-3**

**Figure(J)is the tissue of one control SD rats on the third day respectively.**

**
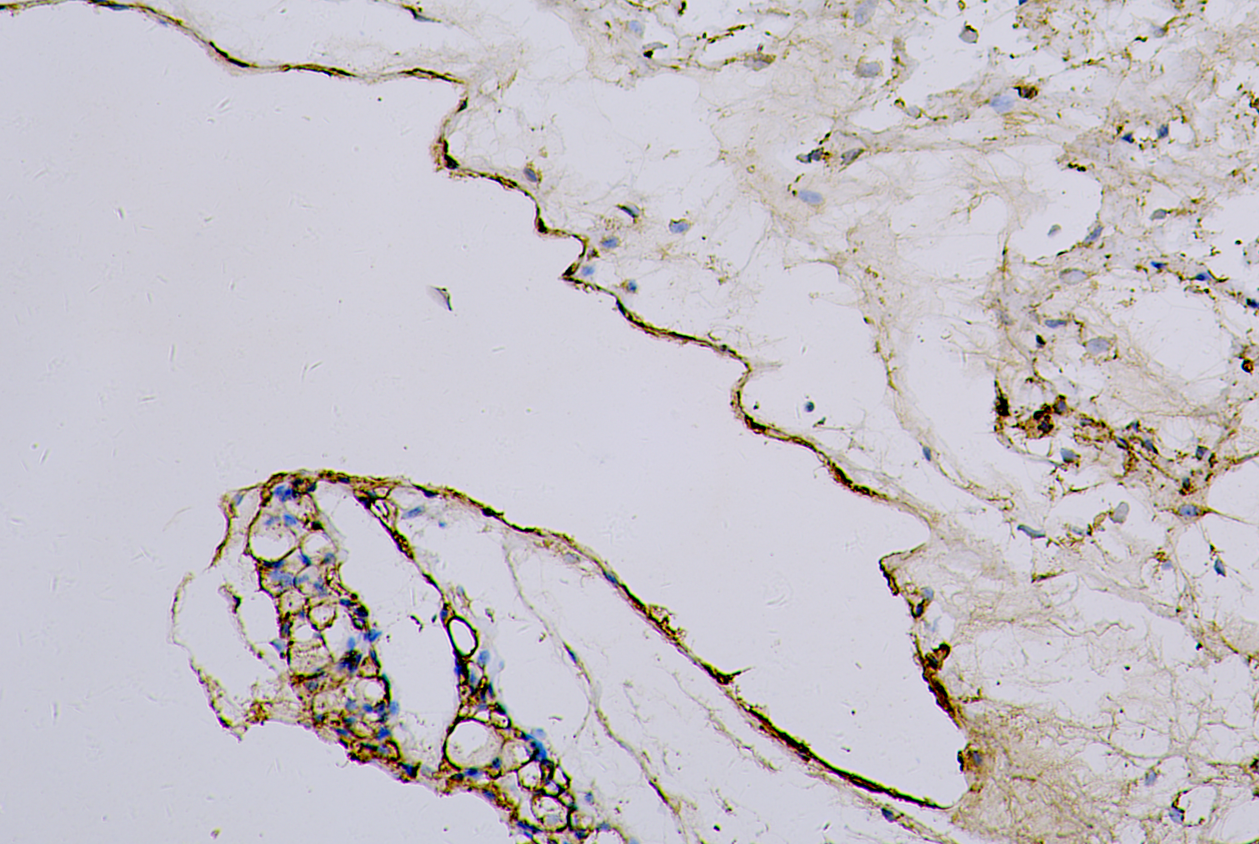
**

**Figure J.****Control-3D-1**

**Figure(K)(L)(M) is the tissue of three DVT SD rats on the seventh day respectively.**

**
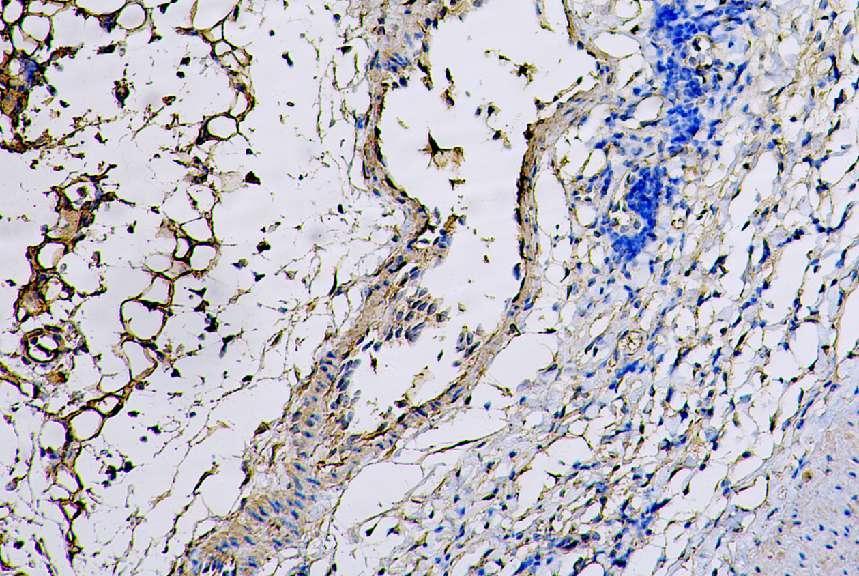
**

**Figure K.DVT-7D-1**

**
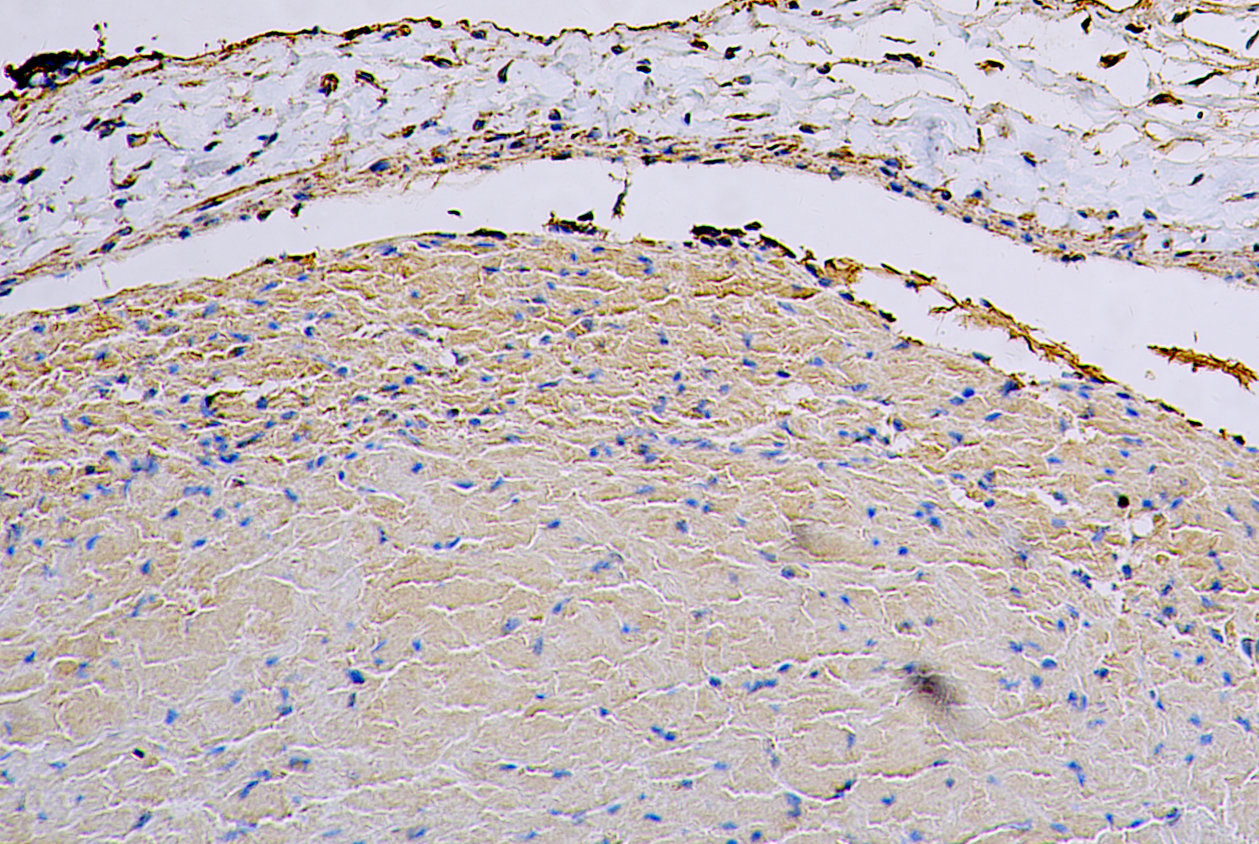
**

**Figure L.DVT-7D-2**

**
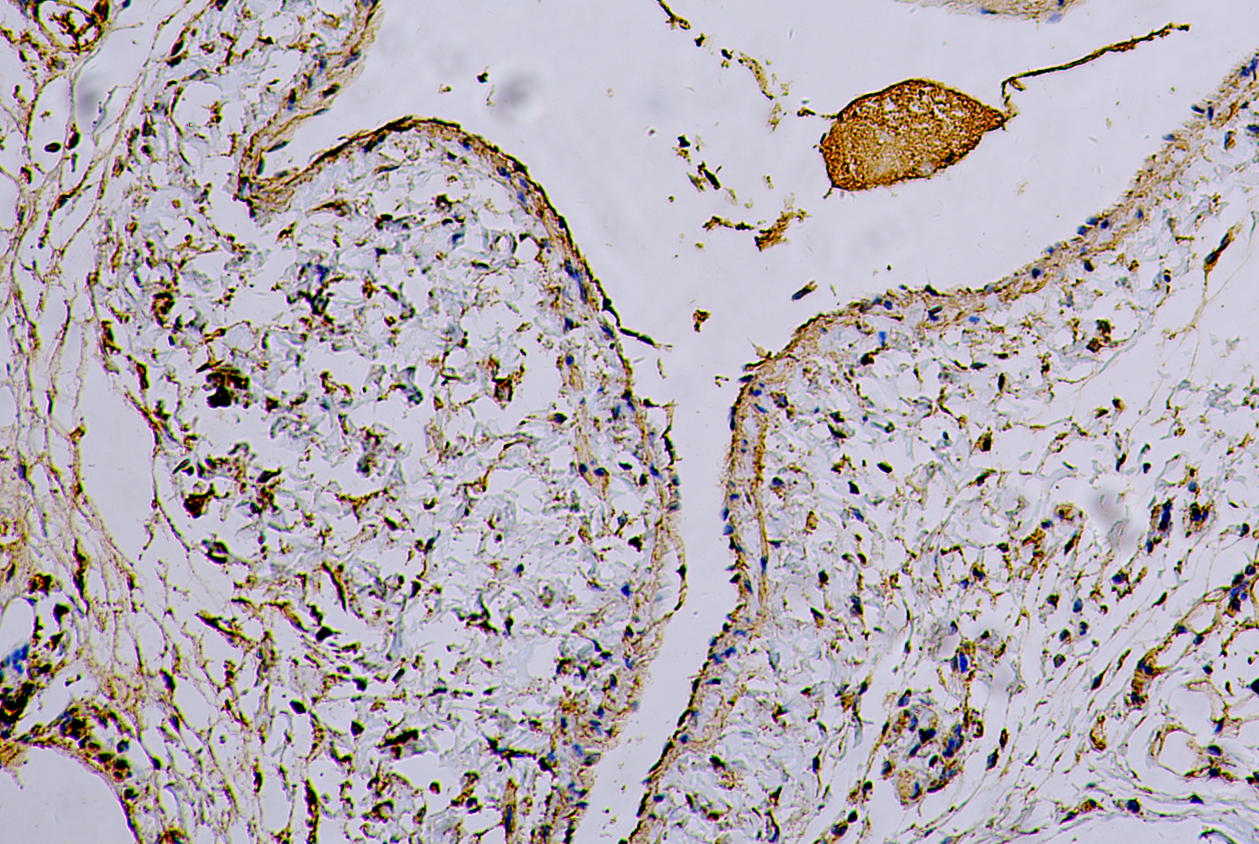
**

**Figure M.DVT-7D-3**

**Figure(N)(O)is the tissue of two control SD rats on the seventh day respectively.**

**
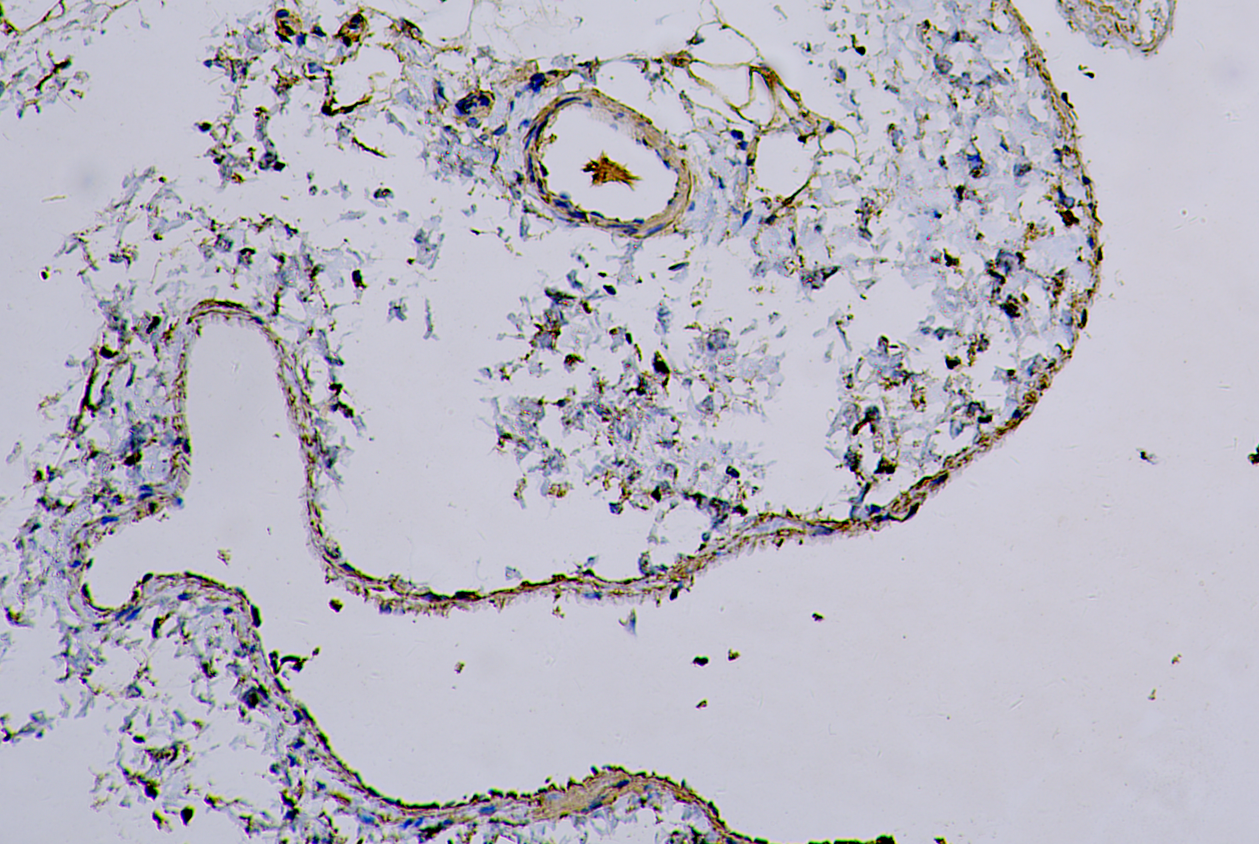
**

**Figure N.Control-7D-1**

**
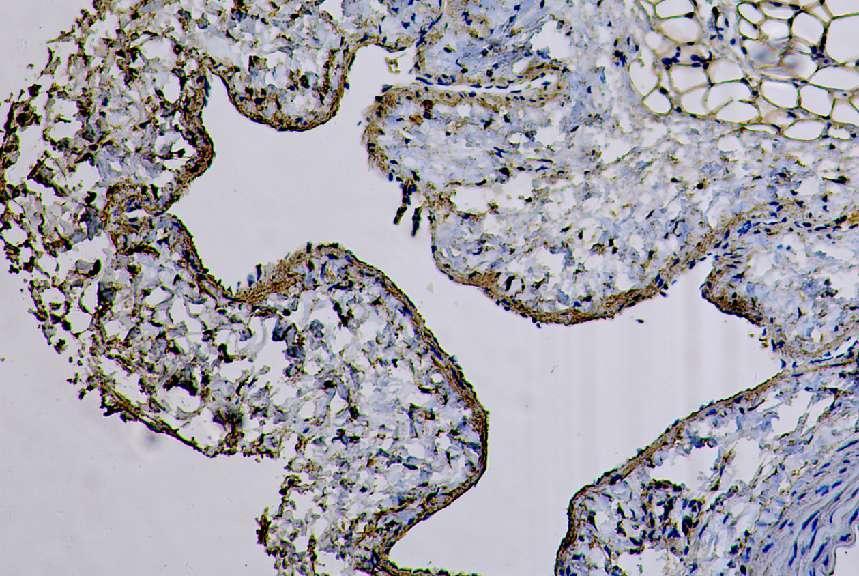
**

**Figure O.Control-7D-2**

**Figure(P)(Q)(R)is the tissue of three DVT SD rats on the** **fourteenth day respectively.**

**
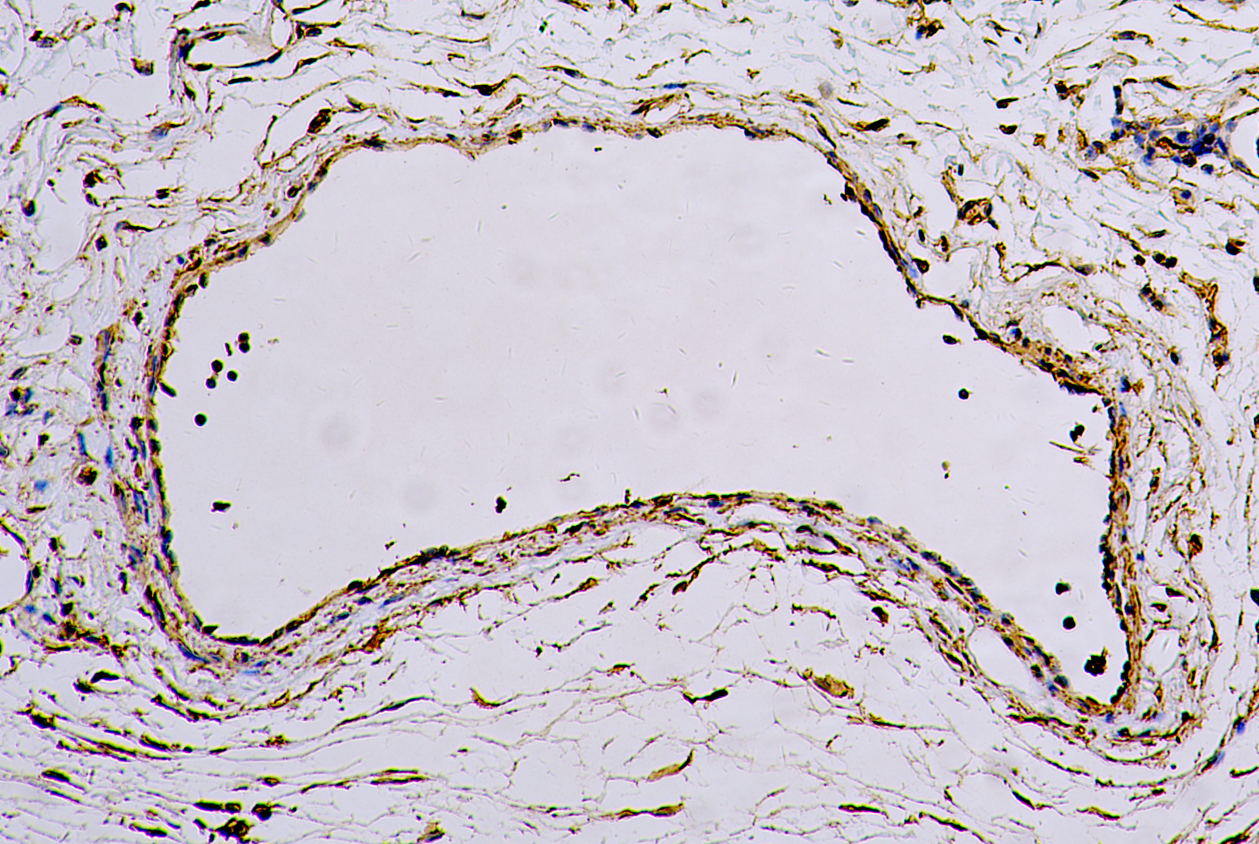
**

**Figure P.DVT-14D-1**

**
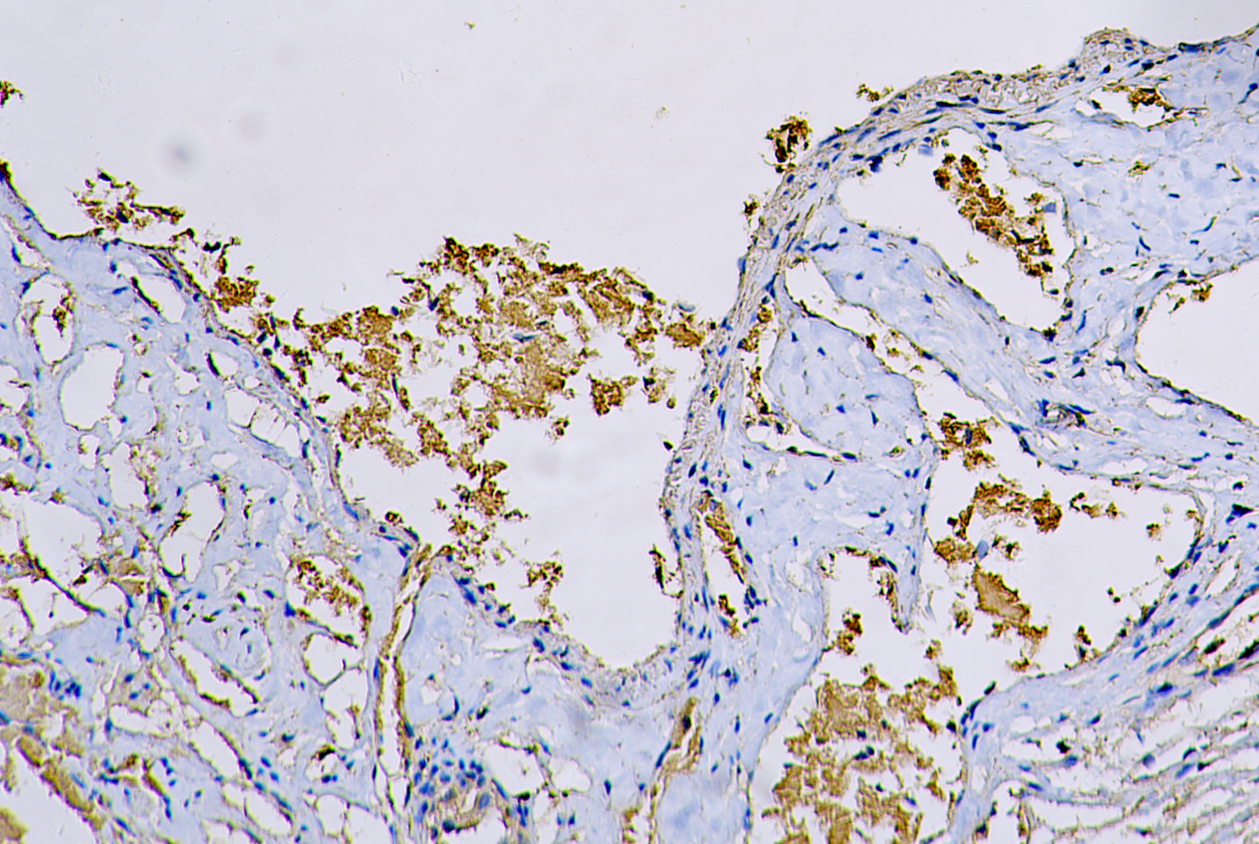
**

**Figure Q.DVT-14D-2**

**
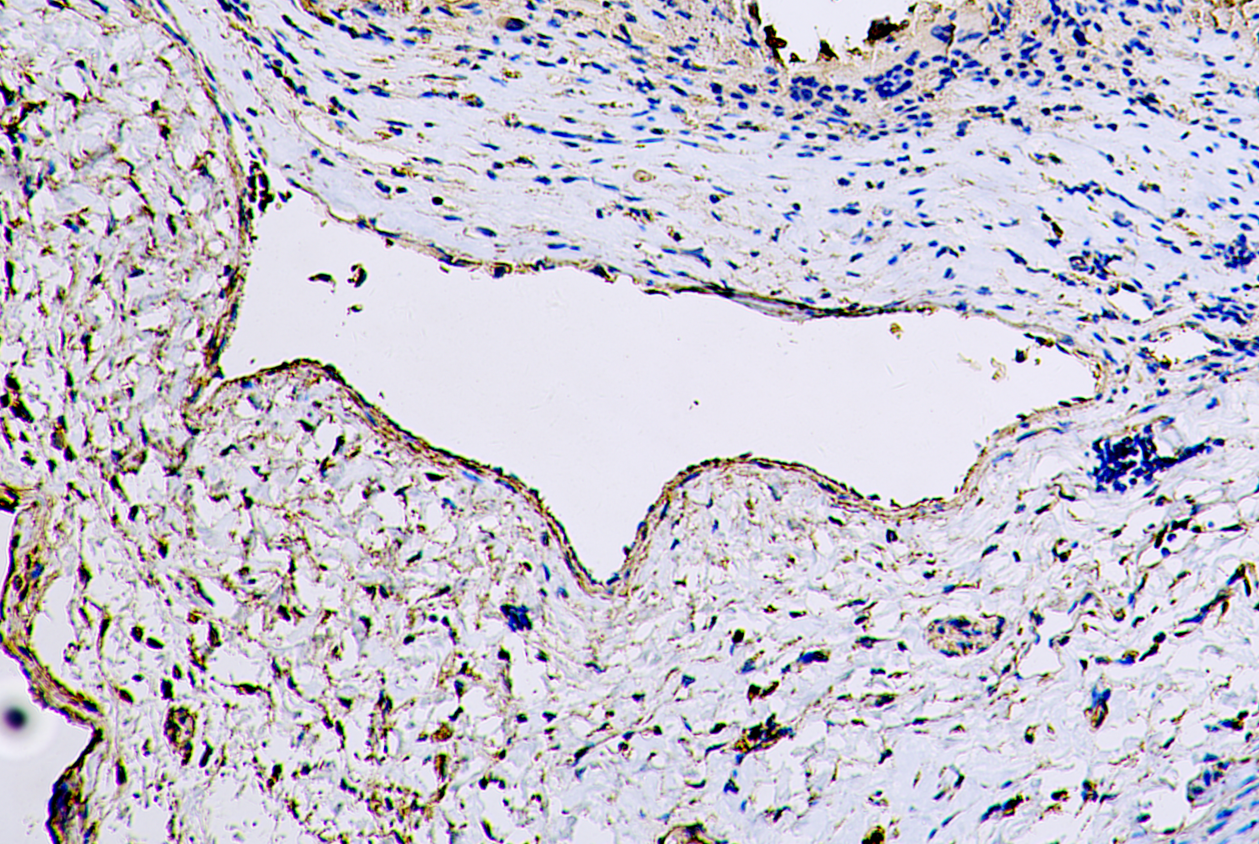
**

**Figure R.DVT-14D-3**

**Figure(S)(T)(U)is the tissue of three control SD rats on the fourteenth day respectively.**

**
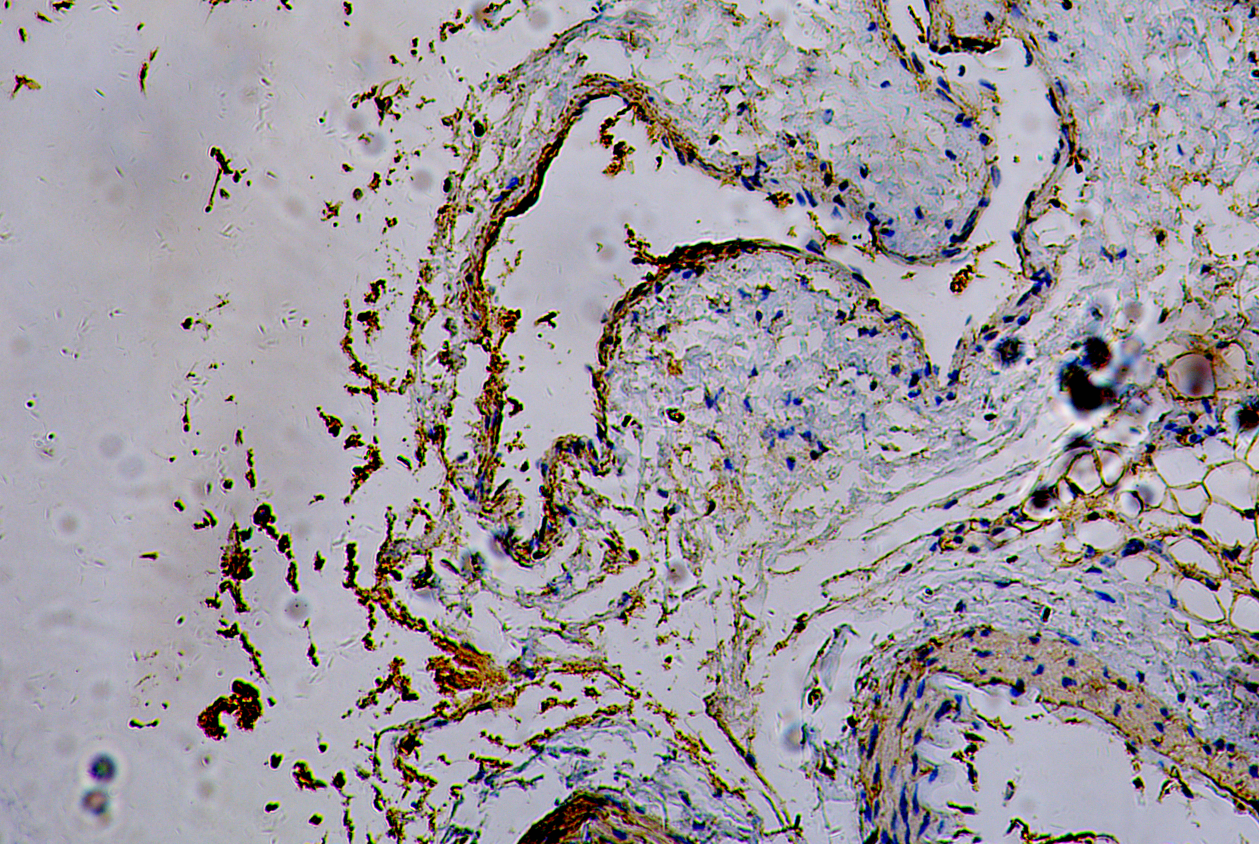
**

**Figure S.****Control-14D-1**

**
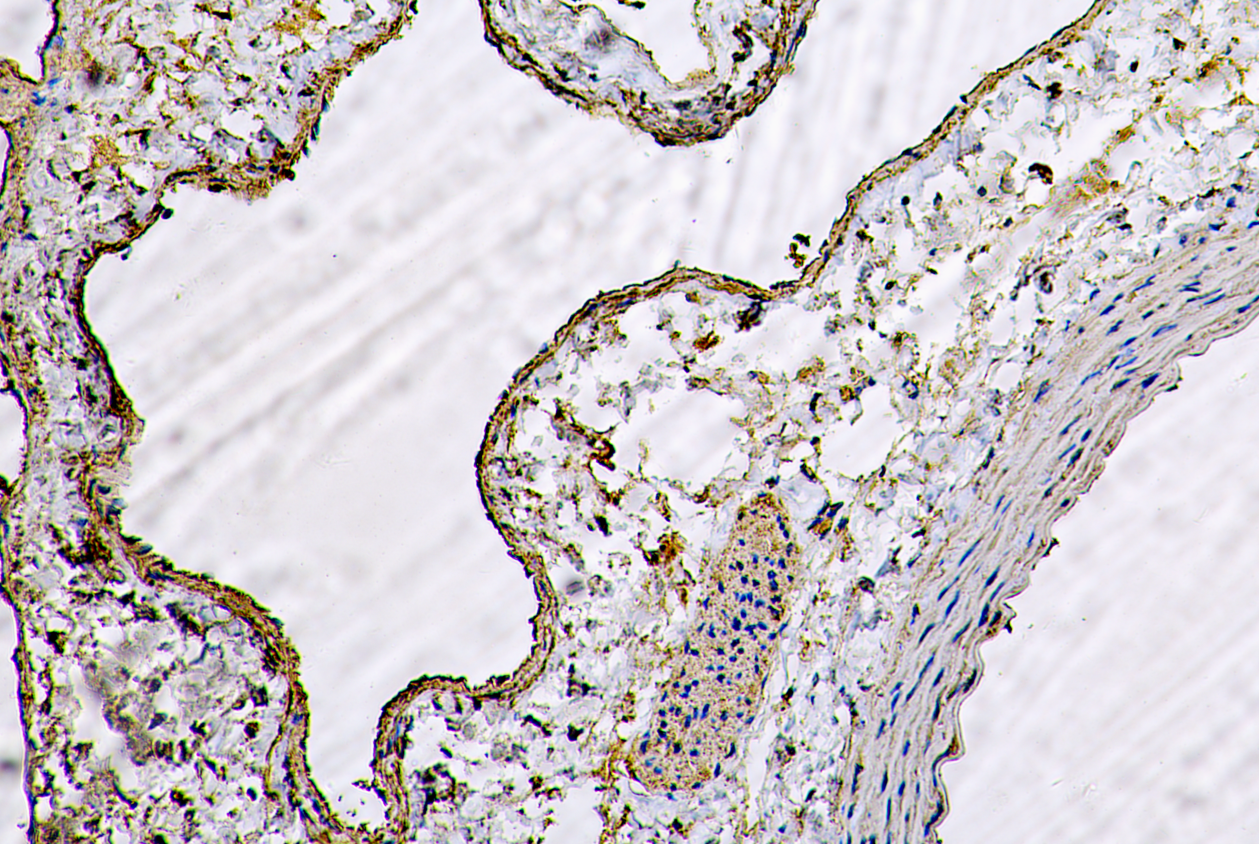
**

**Figure T.Control-14D-2**

**
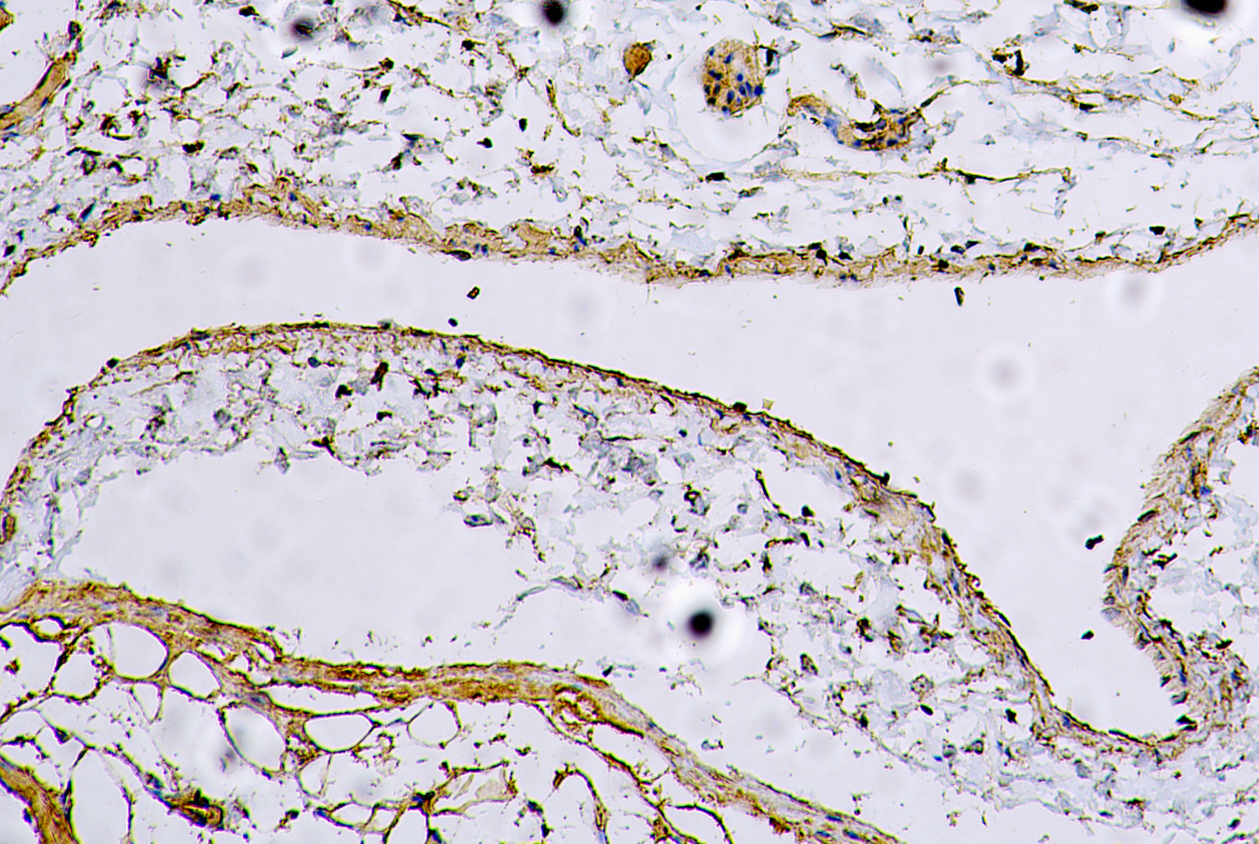
**

**Figure U.Control-14D-3**
