# Supplementary material for: Preliminary clinical analysis and pathway study of S100A8 as a biomarker for the diagnosis of acute deep vein thrombosis
Source: Sci Rep. 2024 Jun 10;14:13298. doi: 10.1038/s41598-024-61728-6 (PMC11164926; doi:10.1038/s41598-024-61728-6)

The following is a numerical record of thrombus thickness, inferior vena cava flow rate, ligation flow rate, and inferior vena cava diameter measured by laser Doppler flowmetry at four time points in DVT rats.


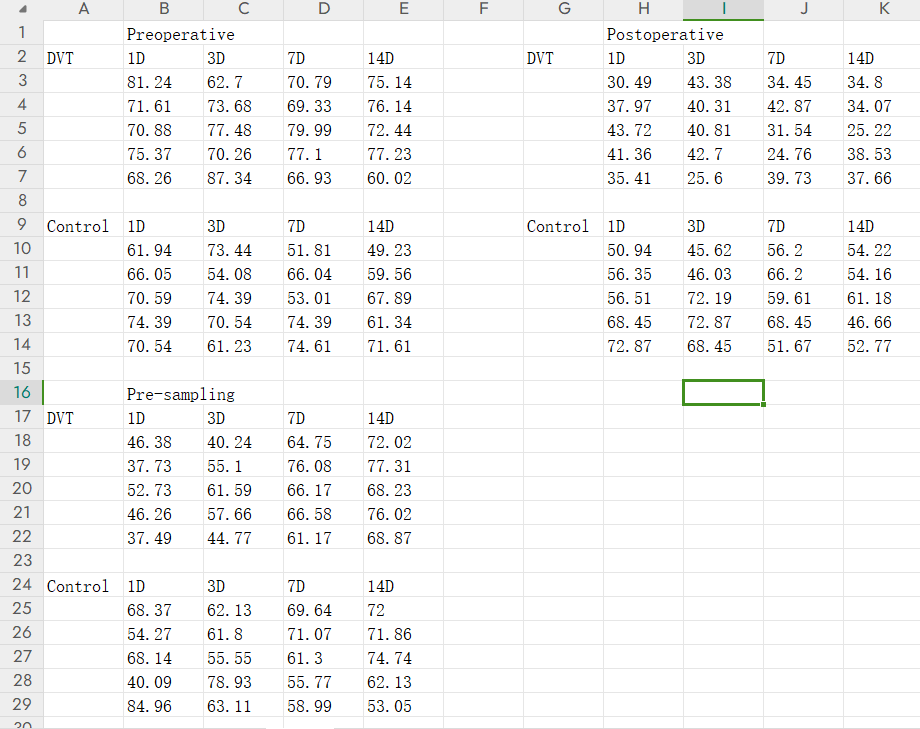


The following is a partial image recording of the thrombus thickness, inferior vena cava flow rate, ligation flow rate, and inferior vena cava diameter of DVT rats measured by Doppler ultrasound on the 1st, 3rd, 7th, and 14 th days.

A(1-3), B, and C(1-4) are thrombus thickness, inferior vena cava flow rate, flow rate at the ligation, and inferior vena cava diameter, respectively, in 3 DVT rats on day 1.


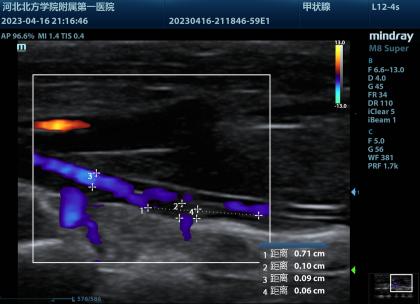

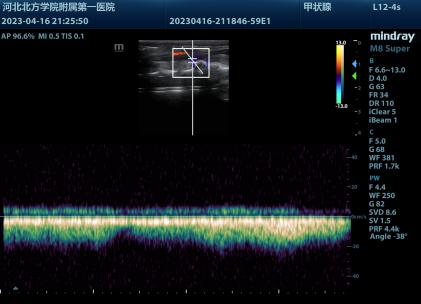

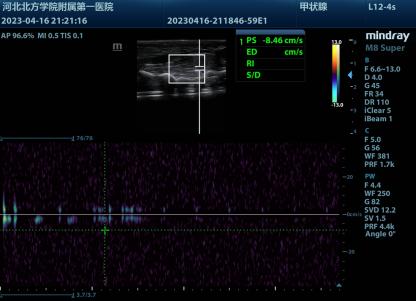


A(1) Thrombus thickness and

inferior vena cava diameter A(2) inferior vena cava flow rate A(3) Flow rate at ligation


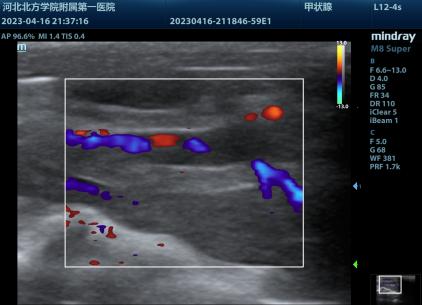

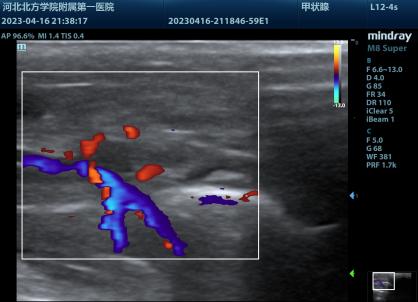

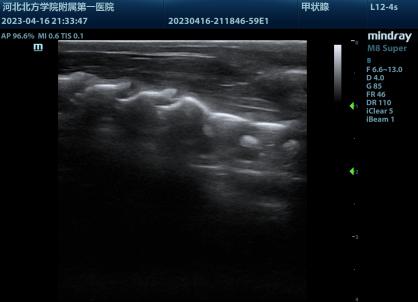


B. These three are from the same DVT rat, but the values are missing from the images; the values were recorded in the experimental record book.


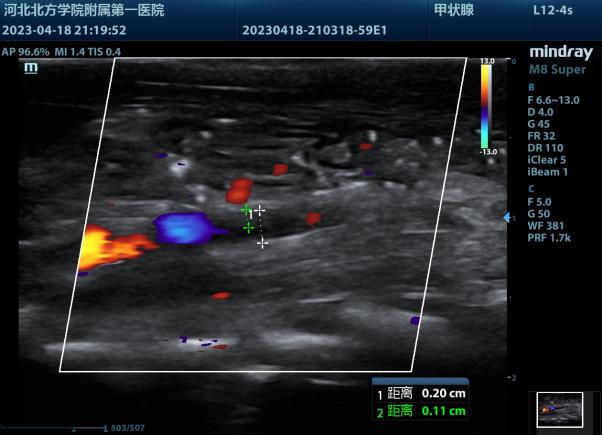

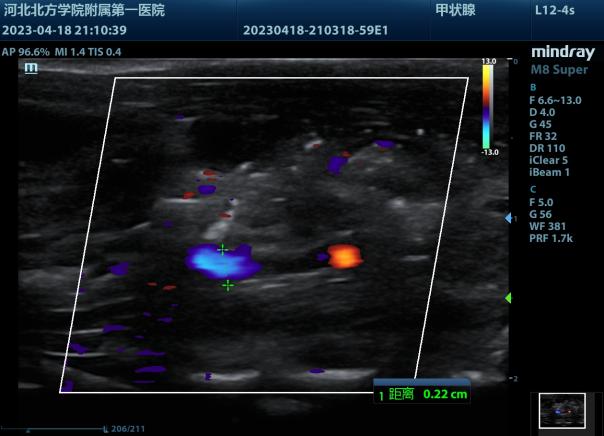


C(1) thrombus thickness C(2) Diameter of inferior vena cava


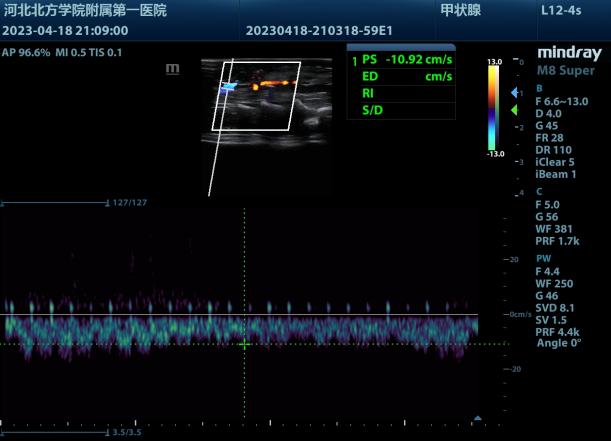

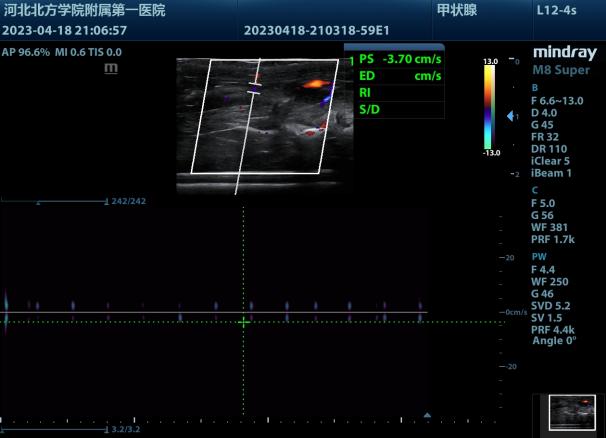


C(3) inferior vena cava flow rate C(4) Flow rate at ligation

D. Thrombus thickness and inferior vena cava diameter in DVT rats on day 3.


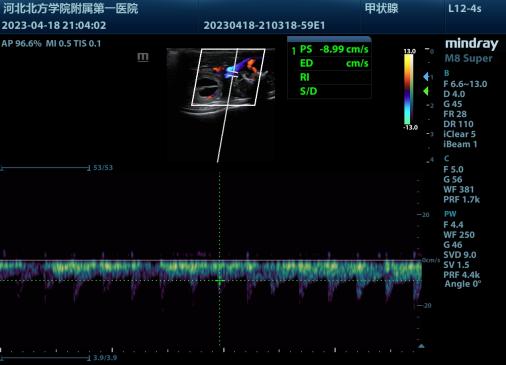

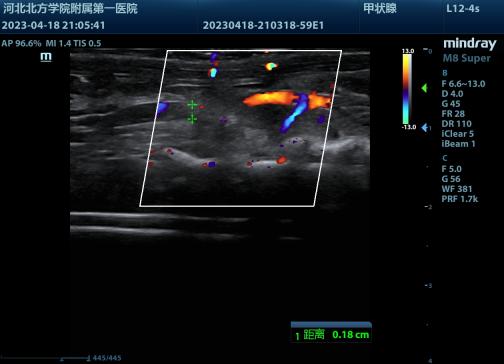


D(1) Inferior vena cava flow rate D(2) Thrombus thickness

E. Thrombus thickness, inferior vena cava flow rate, flow rate at the ligation, and inferior vena cava diameter in DVT rats on day 7.


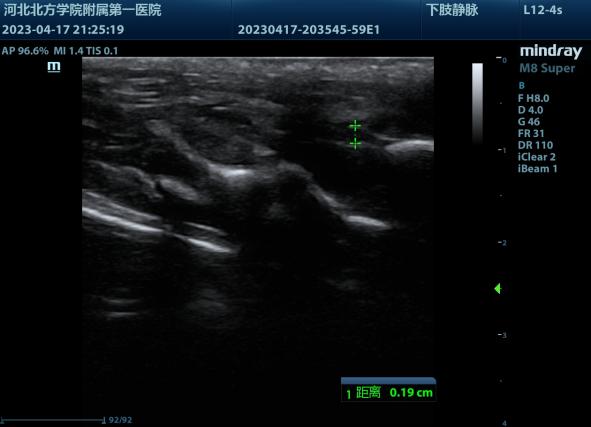

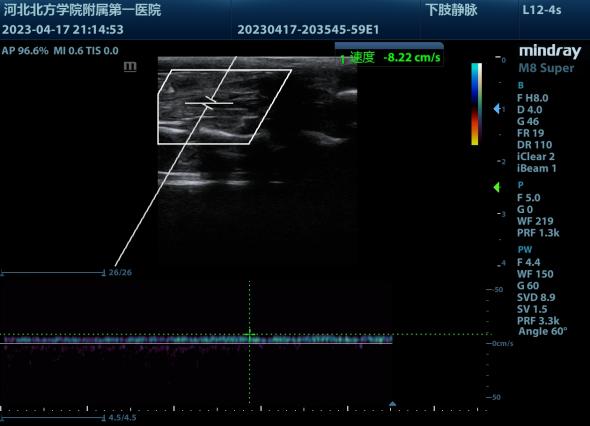


E(1) Thrombus thickness E(2) Inferior vena cava flow rate


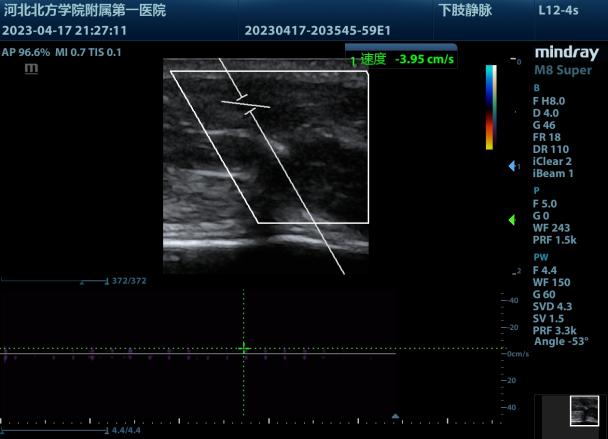

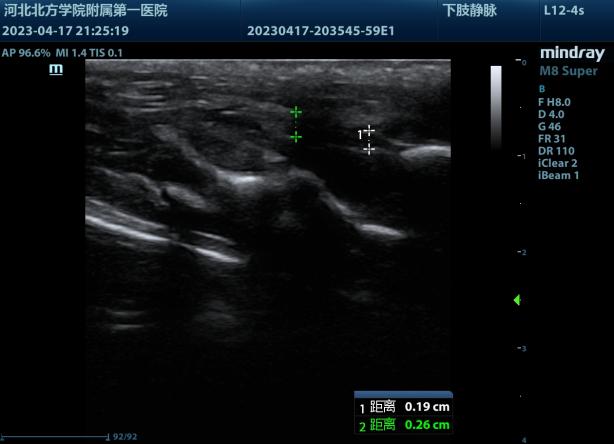


E(3) Flow rate at ligation E(4) Diameter of inferior vena cava

F(1-3), G(1-4), H(1-4), and I(1-4) are thrombus thickness, inferior vena cava flow rate, flow rate at the ligation, and inferior vena cava diameter, respectively, in 4 DVT rats on day 14.


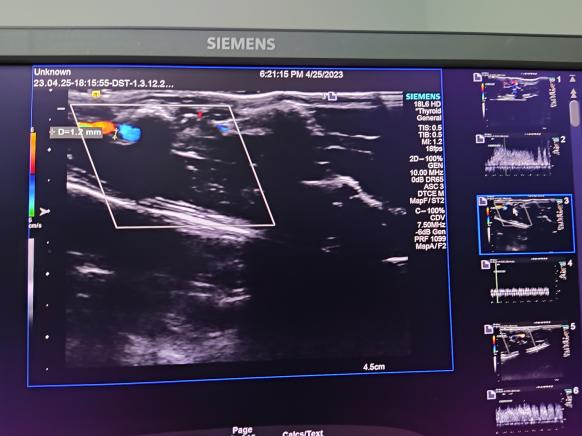

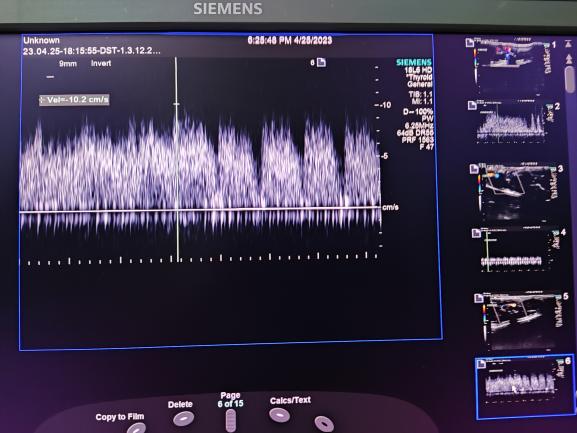


F(1) Thrombus thickness F(2) Inferior vena cava flow rate


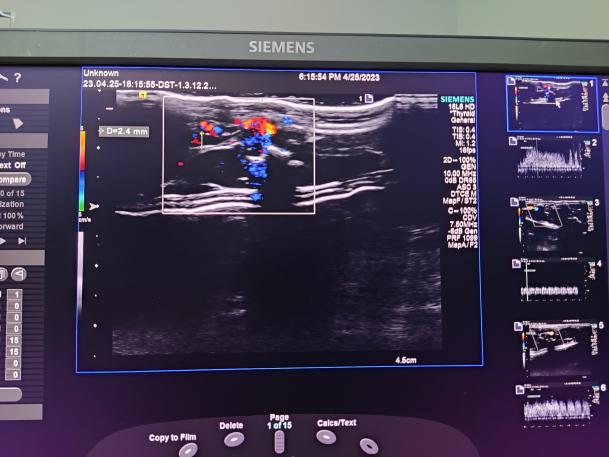


F(3) Diameter of inferior vena cava


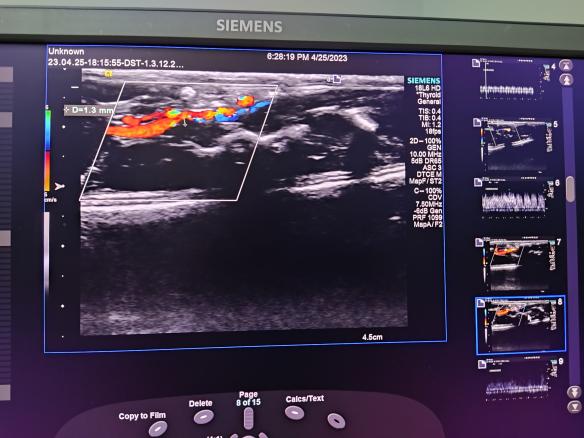

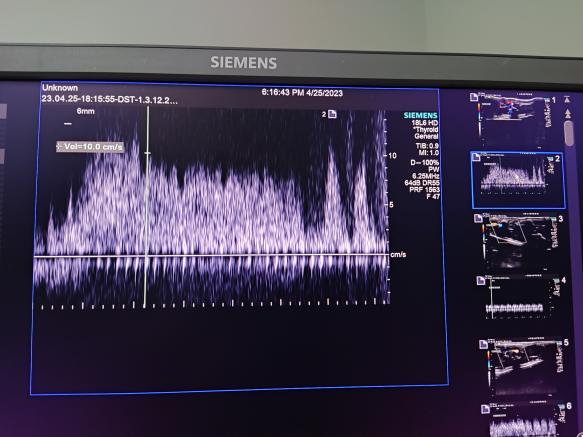


G(1)Thrombus thickness G(2) Inferior vena cava flow rate


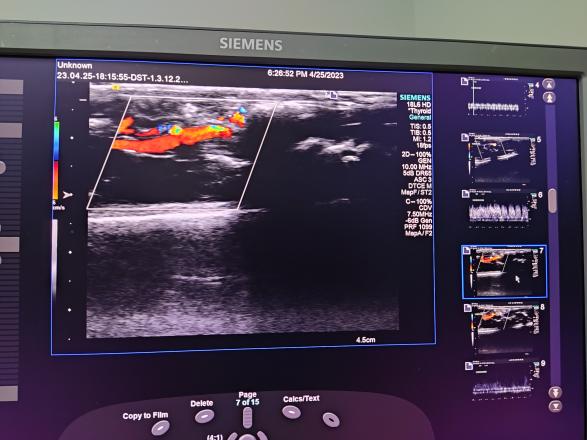

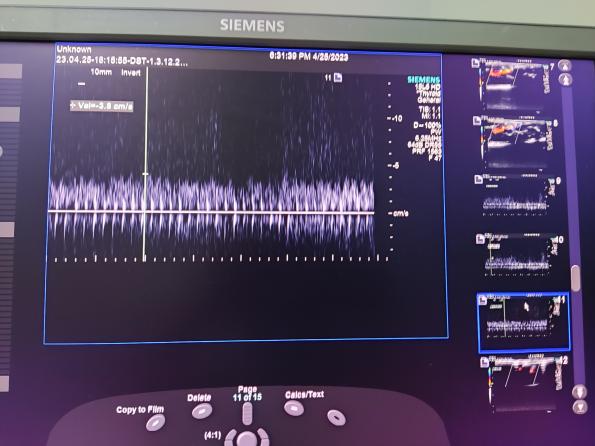


G(3) Diameter of inferior vena cava G(4) Flow rate at ligation


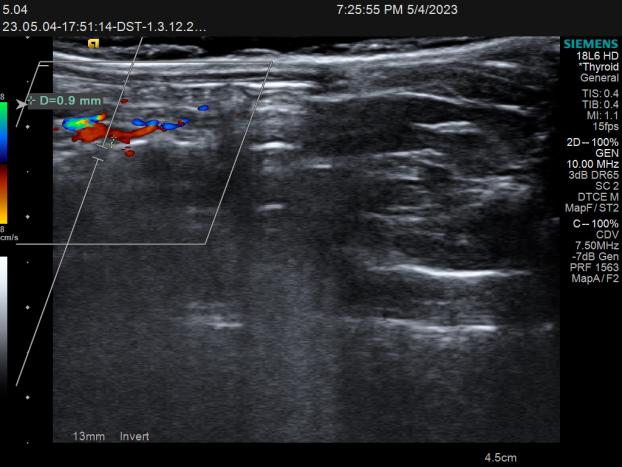

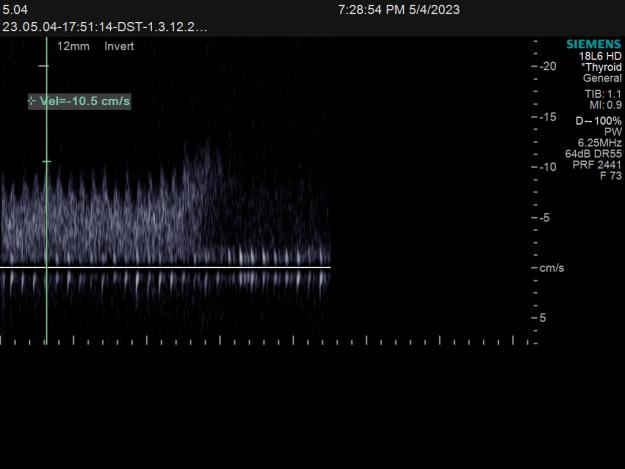


H(1) Thrombus thickness H(2) Inferior vena cava flow rate


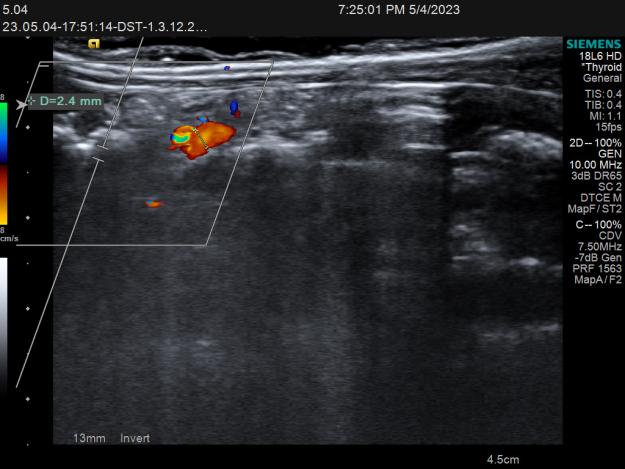

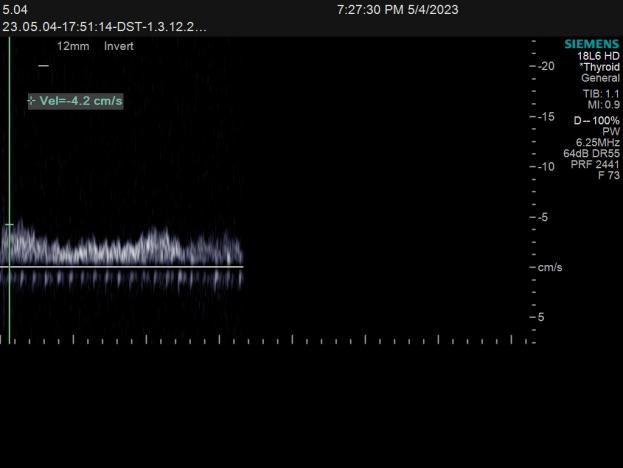


H(3) Diameter of inferior vena cava H(4) Flow rate at ligation


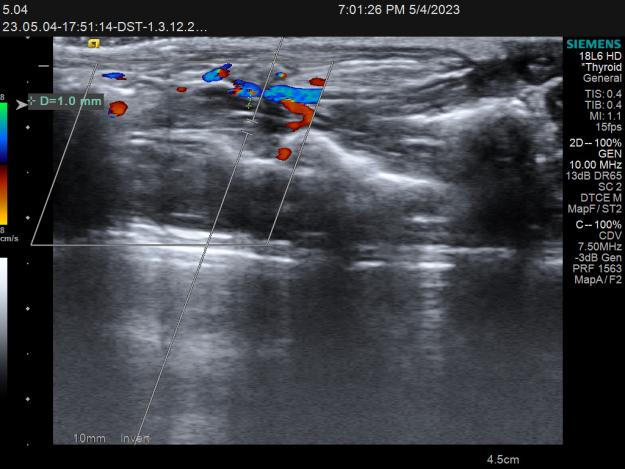

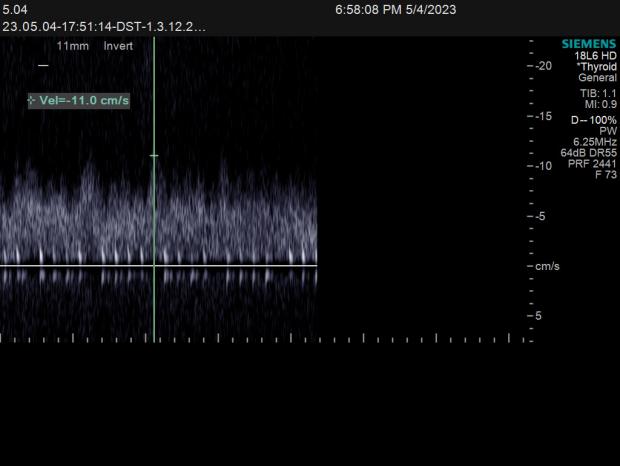


I(1) Thrombus thickness I(2) Inferior vena cava flow rate


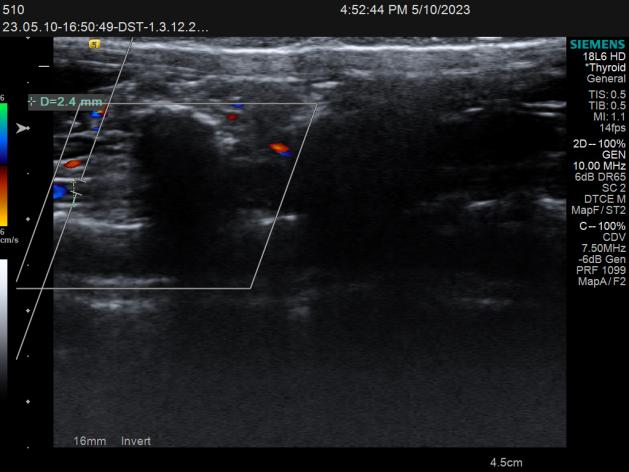

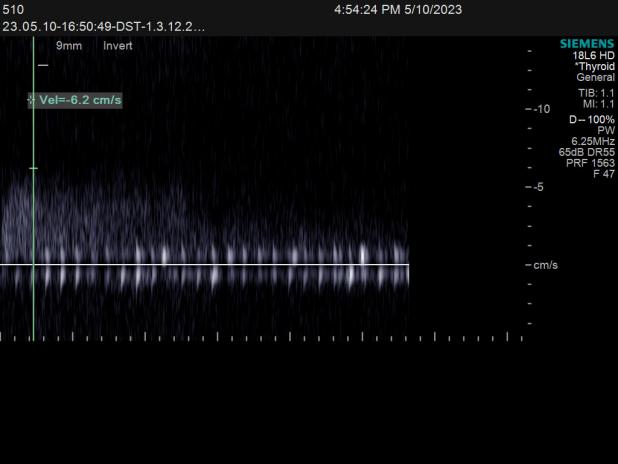


I(3) Diameter of inferior vena cava I(4) Flow rate at ligation

The following is a numerical record of thrombus thickness, inferior vena cava flow rate, ligation flow rate, and inferior vena cava diameter measured by Doppler ultrasound at four time points in DVT rats.


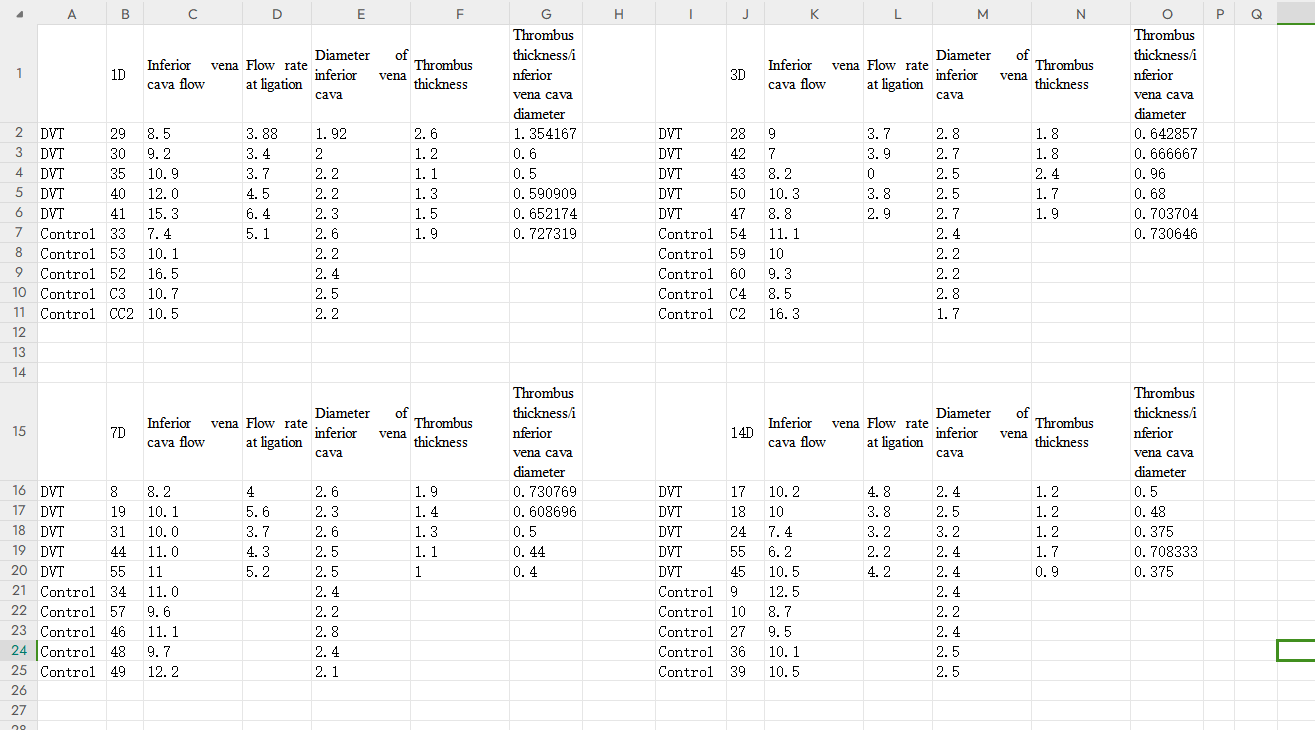

Supplement: Supplementary file 5 — Supplementary Information 5. [file 41598_2024_61728_MOESM5_ESM.docx]
