# Supplementary material for: Preliminary clinical analysis and pathway study of S100A8 as a biomarker for the diagnosis of acute deep vein thrombosis
Source: Sci Rep. 2024 Jun 10;14:13298. doi: 10.1038/s41598-024-61728-6 (PMC11164926; doi:10.1038/s41598-024-61728-6)
Supplement: Supplementary file 6 — Supplementary Information 6. [file 41598_2024_61728_MOESM6_ESM.docx]

The inferior vena cava vascular tissue of DVT rats was measured by vernier caliper on the 1 st, 3 rd, 7 th and 14 th day respectively.

(a)(b)(c) is the tissue of three DVT SD rats on the first day respectively.


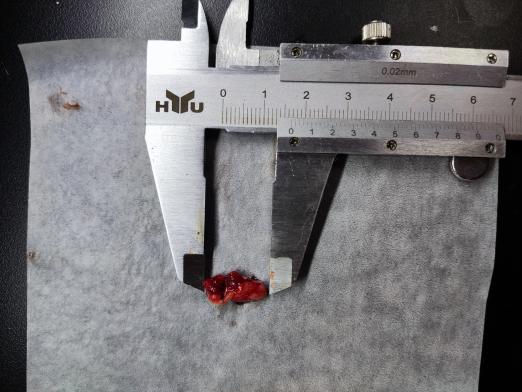

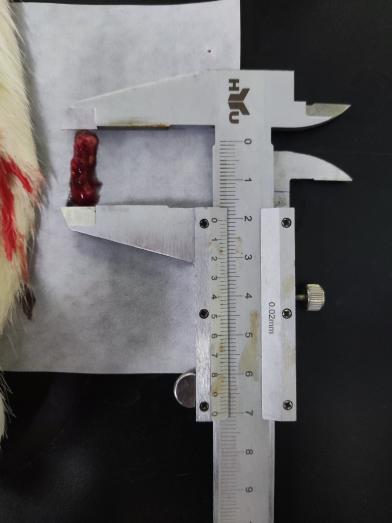

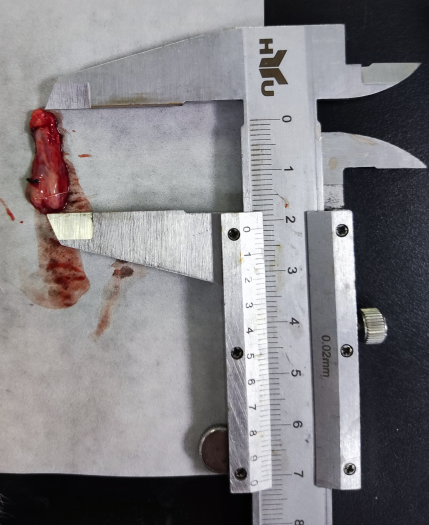


a.DVT-1D-1 b.DVT-1D-2 c.DVT-1D-3

(d)(e)(f) is the tissue of three DVT SD rats on the third day .


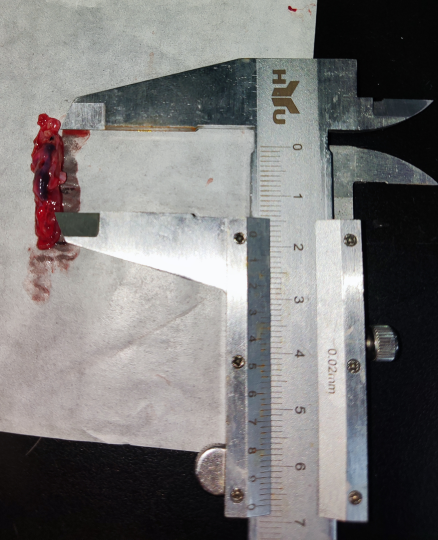

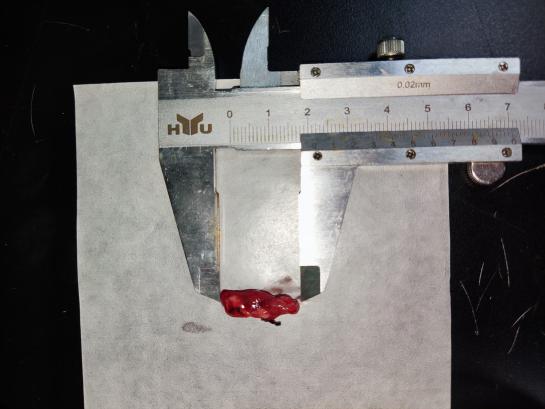

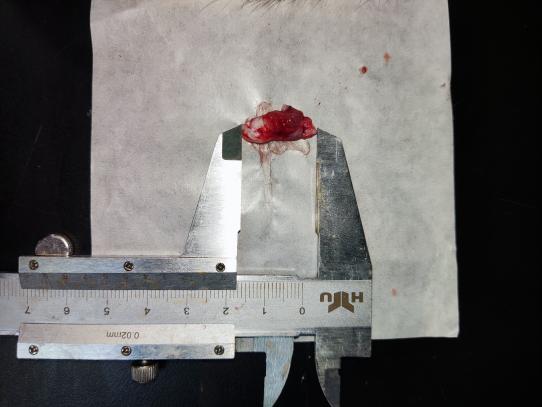


d.DVT-3D-1 e. DVT-3D-2 f.DVT-3D-3

(g)(h)(i) is the tissue of three DVT SD rats on the seventh day respectively.


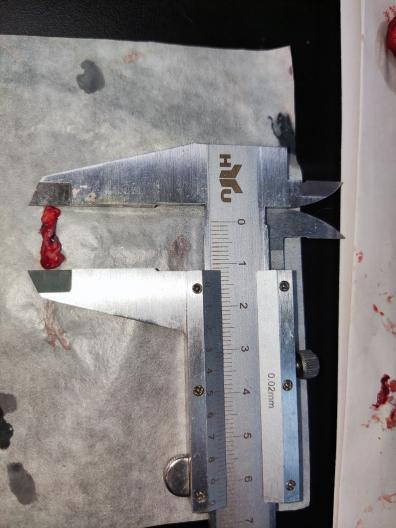

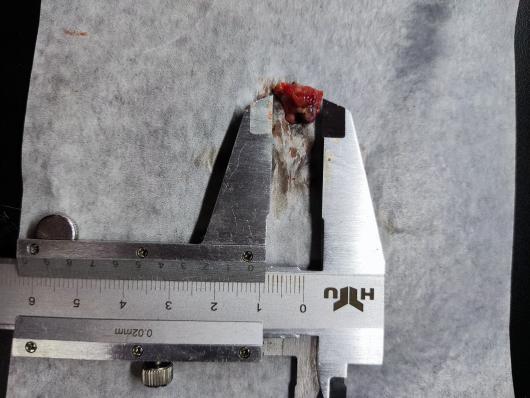

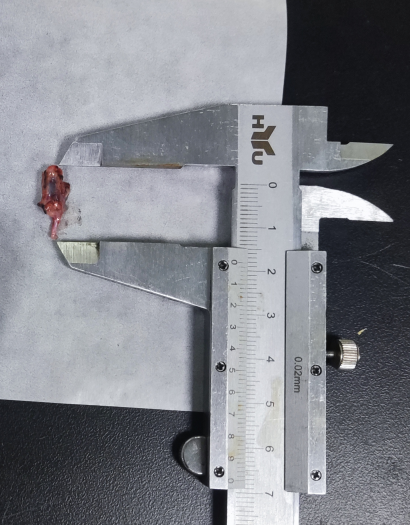


g.DVT-7D-1 h.DVT-7D-2 i.DVT-7D-3

(j)(k)(l) is the tissue of three DVT SD rats on the fourteenth day respectively.


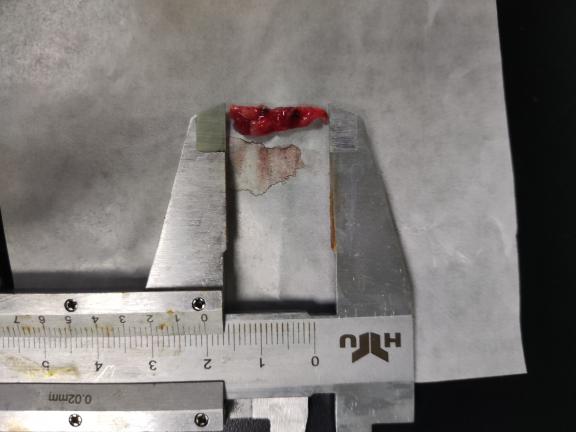

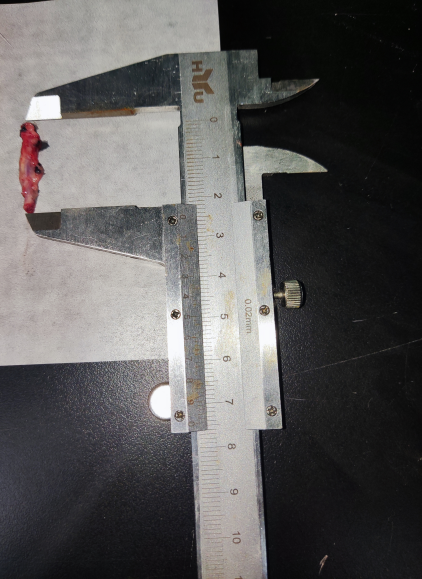

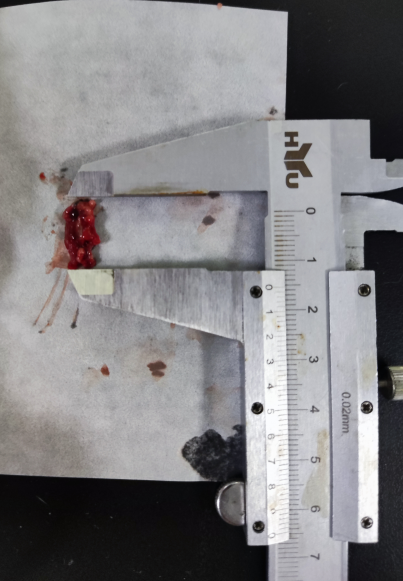


j.DVT-14D-1 k.DVT-14D-2 l.DVT-14D-3
